# Mechanistic insight into the role of mevalonate kinase by a natural fatty acid-mediated killing of *Leishmania donovani*

**DOI:** 10.1038/s41598-022-20509-9

**Published:** 2022-09-30

**Authors:** Surendra Rajit Prasad, Prakash Kumar, Saptarshi Mandal, Anu Mohan, Radhika Chaurasia, Ashish Shrivastava, Pallaprolu Nikhil, Dande Aishwarya, P. Ramalingam, Rahul Gajbhiye, Shriya Singh, Arunava Dasgupta, Mukesh Chourasia, V. Ravichandiran, Prolay Das, Debabrata Mandal

**Affiliations:** 1 Department of Biotechnology, National Institute of Pharmaceutical Education and Research, Export Promotions Industrial Park (EPIP), Vaishali District, Hajipur, Bihar 844102 India; 2 Department of Chemistry, Indian Institute of Technology, Patna Bihta, Bihar 801106 India; 3 Translational Bioinformatics and Computational Genomics Research Lab, Department of Life Sciences, Shiv Nadar University, G.B. Nagar, Uttar Pradesh 201314 India; 4 Department of Pharmaceutical Analysis, National Institute of Pharmaceutical Education and Research, Hajipur, 844102 India; 5 Molecular Microbiology and Immunology Division, CSIR-Central Drug Research Institute, Sitapur Rd, Sector-10, Jankipuram Extension, Lucknow, Uttar Pradesh 226031 India; 6 Academy of Scientific and Innovative Research (AcSIR), Ghaziabad, 201002 India; 7 Amity Institute of Biotechnology, Amity University, Sector 125, Noida, Uttar Pradesh 201301 India; 8 National Institute of Pharmaceutical Education and Research, Kolkata, 700054 India

**Keywords:** Biochemistry, Computational biology and bioinformatics, Microbiology, Molecular biology

## Abstract

We evaluated the anti-leishmanial efficacy of different saturated medium-chain fatty acids (FAs, C8–C18) where FA containing C8 chain, caprylic acid (CA), was found to be most potent against *Leishmania donovani,* the causative agent for visceral leishmaniasis (VL). Different analogs of CA with C8 linear chain, but not higher, along with a carboxyl/ester group showed a similar anti-leishmanial effect. Ergosterol depletion was the major cause of CA-mediated cell death. Molecular docking and molecular dynamic simulation studies indicated the enzyme mevalonate kinase (MevK) of the ergosterol biosynthesis pathway as a possible target of CA. Enzyme assays with purified recombinant MevK and CA/CA analogs confirmed the target with a competitive inhibition pattern. Using biochemical and biophysical studies; strong binding interaction between MevK and CA/CA analogs was established. Further, using parasites with overexpressed MevK and proteomics studies of CA-treated parasites the direct role of MevK as the target was validated. We established the mechanism of the antileishmanial effect of CA, a natural product, against VL where toxicity and drug resistance with current chemotherapeutics demand an alternative. This is the first report on the identification of an enzymatic target with kinetic parameters and mechanistic insights against any organism for a natural medium-chain FA.

## Introduction

Leishmaniasis is one of the twenty major neglected tropical diseases (NTDs) listed by the World Health Organization (WHO) and the second-largest protozoal disease after malaria^1^. This disease affects the lives of around 12 million people in 98 countries by causing mainly self-healing cutaneous lesions (CL) to severe and systemic visceral leishmaniasis (VL). The VL, which is also known as Kala-azar, is caused by *Leishmania donovani* (LD) that threatens an estimated 500,000 new cases per year mostly in East Africa and Indian subcontinent with an estimated 70,000 mortalities per year^2^. Although pentavalent antimonials and sodium antimony gluconate (SAG) are supposedly the first lines of medications practiced in the last two decades of the twentieth century, widespread drug resistance against them has been observed. Conventional chemotherapy is currently prevalent and mostly depends on Amphotericin B (AmB) as deoxycholate (fungizone) or AmB-liposomal (AmBisome) formulation to date. However, severe nephrotoxicity became a problem for fungizone whereas the high cost of AmBisome treatment creates a burden for effective and prolonged treatment of VL^3,4^.

The emergence of human immunodeficiency virus-VL (HIV-VL) co-infections and post-kala-azar dermal leishmaniasis (PKDL) which emerges as a skin-lesion post-VL treatment generates a new sociological problem related to the eradication of the disease by 2020 as proposed by WHO^5^. Further, the lack of interest in R&D of private firms due to low revenue in treating a poorer class of people in 3rd world countries is not favoring the development of new therapies for effective treatment of VL. The development of novel and low-cost potential antileishmanial agents from natural resources may help in the management of a NTD like VL^6^. Different plant extracts, phytochemicals, and purified natural products were tested against leishmaniasis either by using directly or in different nanoformulations with antileishmanial efficacy^7–9^. The obvious advantage is the low cost in development and reduced toxicity compared to currently used chemotherapeutics.

Since FAs with a chain length greater than the C12 backbone are generally part of a biological membrane, FAs and their derivatives with medium chain length (C6–C12) were explored as antimicrobial agents long back^10–12^. Among these FAs, caprylic acid (CA, C8) and lauric acid (LA, C12) were explored, extensively, as antifungal and antibacterial agents^13–16^. CA and LA are naturally present and abundant in coconut oil/cream and the breast milk of humans. These naturally occurring FAs and most of their derivatives are Generally Recognized As Safe (GRAS) reagents by the United States Food and Drug Administration (USFDA) and other regulatory agencies. The primary mode of antifungal action of CA involves inhibition of biofilm formation^17^ or disruption of membrane integrity^18^. The membrane damaging effect of CA against yeast and bacteria was also reported^19,20^. The derivatives of CA were also found to be antibacterial^21^ and, even, effective against drug-resistant fungal infections^22^. Monoglycerides of CA are extensively used as preservatives in many formulations and cosmetic applications^23^. However, to date, there is no report of CA and its derivatives as antiparasitic or antileishmanial agents. We, report here for the first time the antileishmanial effect and underlying molecular mechanism of action of CA against LD. CA kills the parasite by depleting ergosterol and inhibiting the enzyme mevalonate kinase (MevK) of the sterol biosynthesis pathway. CA and its derivatives, with a C8 backbone, act as a competitive inhibitor of MevK by mimicking the structure of the substrate R-mevalonate. Ergosterol (ERG) depletion observed in CA-treated cells along with molecular docking and molecular dynamics (MD) simulation studies hinted at the enzyme MevK as a plausible target for CA. Further, enzyme assay with recombinant MevK enzyme, isothermal calorimetry (ITC) analysis for binding interactions between MevK-CA, proteomics studies of CA-treated parasites, and over-expression of MevK in LD cells confirms the role of MevK as the molecular target for CA.

## Results

### Caprylic acid and its analogs with C8 linear chain are most effective against LD promastigotes and amastigotes

**Figure 1:**
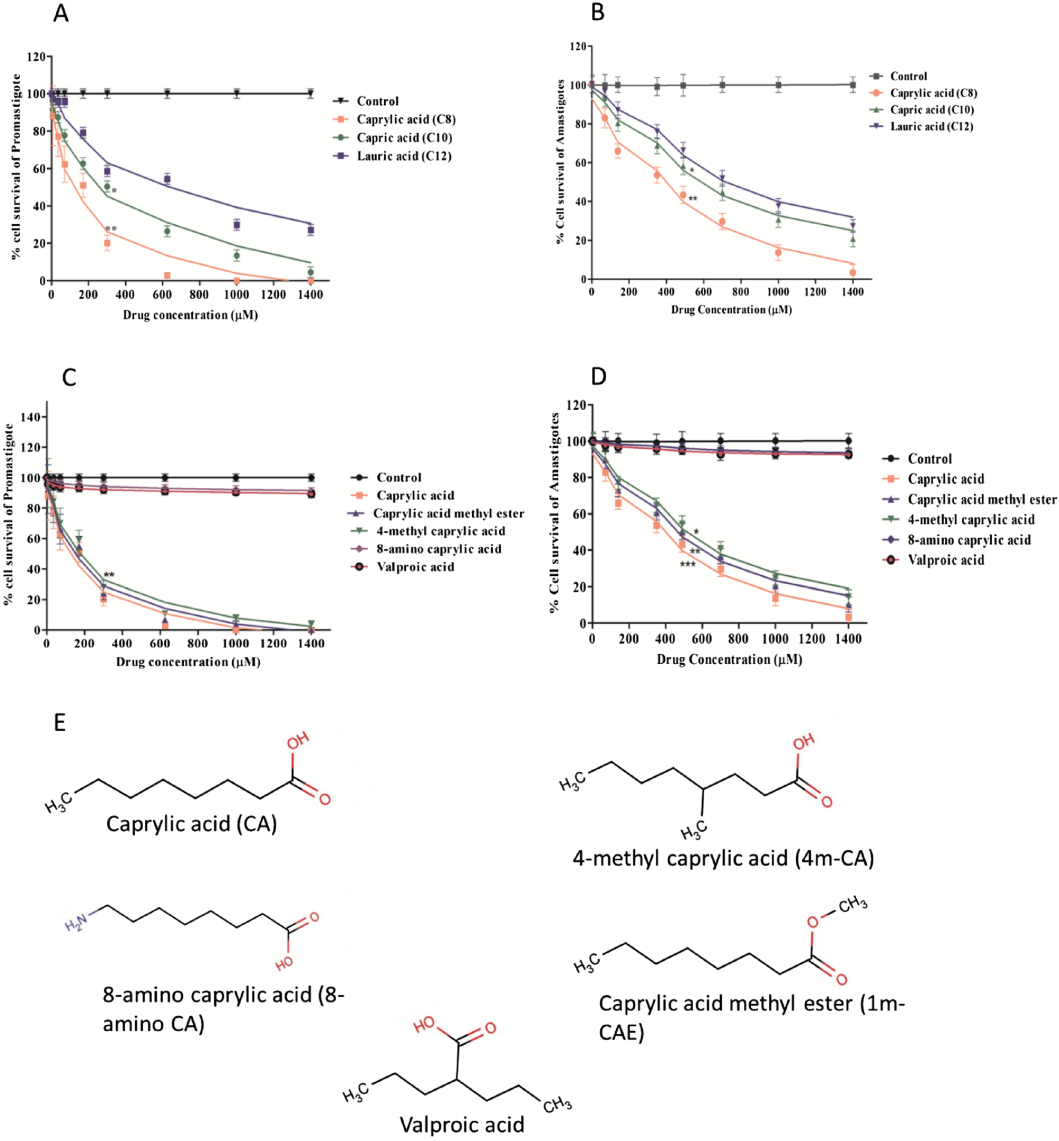
The anti-leishmanial activity of CA, capric acid and lauric acid after 48 h treatment against *L. donovani* promastigotes in vitro (**A**) and against intracellular amastigotes ex vivo (**B**). Further, the anti-leishmanial activity of caprylic acid methyl ester (1m-CAE), 4-methyl caprylic acid (4m-CA), 8-amino caprylic acid and valproic acid were compared with caprylic acid for both promastigotes (**C**) and amastigotes (**D**) at the same time interval. Structure of CA and their analogues used in this study (**E**). Note: *P < 0.05; **0.05 < P < 0.01; ***0.01 < P < 0.001.

The antileishmanial efficacy of saturated medium-chain FAs (C8–C18) was tested in a dose- and time-dependent manner against promastigotes. After 48 h the IC_50_ for CA is ~ 173 µM, (~ 25 µg/ml, P < 0.01), whereas for capric acid (CRA) and LA the IC_50_ is ~ 310 µM (~ 45 µg/ml) and ~ 450 µM (~ 95 µg/ml), respectively (Fig. 1A). However, FAs with longer chain length (C14–C18) have no or minimal effect on the killing of the parasite (Fig. S1A). Therefore, CA with the C8 chain has the maximum antileishmanial effect than any other saturated FAs tested here.

For amastigote-infected macrophages, after 48 h the IC_50_ for CA is ~ 372 µM (P < 0.001) whereas for CRA and LA the IC_50_ is ~ 625 µM and ~ 730 µM (Fig. 1B). The IC_50_ for CA after 72 h is ~ 150 µM and ~ 350 µM against promastigotes and amastigotes, respectively (Fig. SI-2A and 2B). A nearly ~ 2.2 fold increase in IC_50_ value of CA for amastigotes compared to promastigotes is possibly due to reduced uptake through macrophage membranes.

Since FAs with a C8 backbone are showing the highest efficacy we evaluated the antileishmanial efficacy of different analogs of the C8 chain (8-amino CA, valproic acid, 4 methyl CA and caprylic acid methyl ester). Interestingly, 4-methyl CA (4m-CA) and caprylic acid methyl ester (1m-CAE) show almost the same efficacy as CA but 8-amino CA and valproic acid have significantly reduced efficacy (Fig. 1C). Also, 4m-CA and 1m-CAE showed almost similar effects to CA against amastigote-infected parasites (Fig. 1D). Therefore, the presence of the C8 linear chain and carboxylate/ester group at C1 position is found to be essential for the antileishmanial efficacy of C8 derivatives. Also, branching of FA with –COOH group in the middle (valproic acid) or inclusion of a charged group like –NH_2_ (8-amino CA) in the linear C8 chain causes loss of antileishmanial effect. However, an alkyl substitution at C4 (4m-CA) or C1 (1m-CAE) maintains the same level of efficacy in comparison with CA indicating further the necessity of a linear C8 chain and a terminal carboxylate/ester group in parasite killing (Fig. 1E).

### Cytotoxicity and hemolytic activity of CA

**Figure 2:**
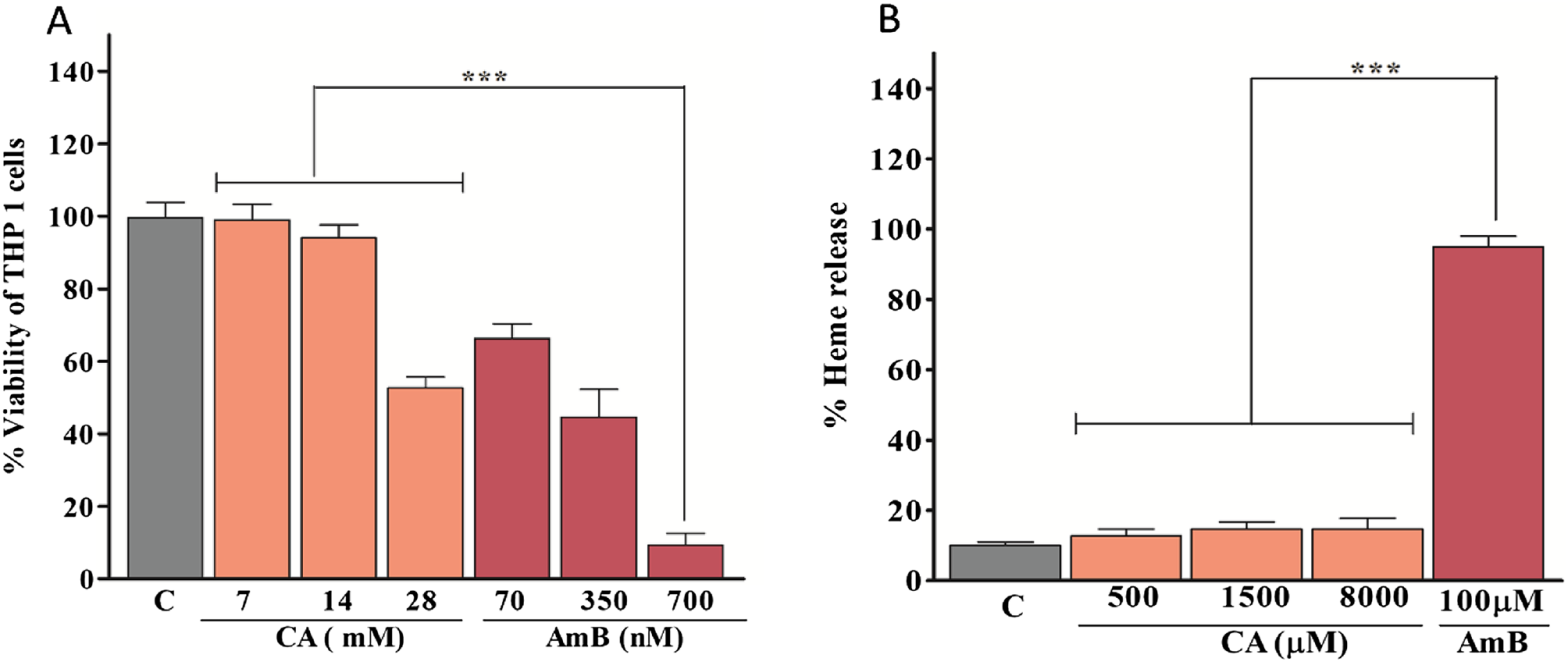
Cytotoxicity was evaluated on the THP-1 and RBCs cells for caprylic acid and AmB. Cytotoxicity assay on THP-1 cells after 48 h treatment with caprylic acid and AmB (**A**). Hemolysis assay on human red blood cells after 4 h treatment with caprylic acid and AmB (**B**). Comparative efficacy of CA and AmB in cytotoxicity is shown in these graphs. Note: ***0.01 < P < 0.001.

Since CA has an IC_50_ of ~ 173 µM against promastigotes, which is higher compared to other conventional antileishmanial agents like AmB, miltefosine, or pentamidine we are interested to know its cytotoxicity against human monocytic THP-1 cells. For CA, the CC_50_ (concentration showing 50% killing of monocytes compared to untreated control) after 48 h was ~ 28 mM which is > 75-fold higher than the IC_50_ (~ 372 µM) against amastigote infected macrophages (Fig. 2A). Further, with 14 mM of CA (> 37 fold higher than IC_50_ against amastigote infected macrophages), the killing of human monocytes is only ~ 6%. For AmB-deoxycholate (which is used as a positive control) the CC_50_ value was ~ 0.31 µM. Therefore, CA is significantly less cytotoxic than AmB (Fig. 2A, P < 0.001). Further, CA was found to be less haemolytic than AmB-deoxycholate. At a 100 μM dose, AmB showed ~ 95% hemolysis but CA showed only ~ 12% hemolysis even at 500 μM (P < 0.001). At an 8 mM concentration of CA, the hemolytic activity was ~ 14% (Fig. 2B). Therefore, CA is less cytotoxic and hemolytic than AmB-deoxycholate.

### Caprylic acid-treated cells show membrane damage with LDH release

**Figure 3:**
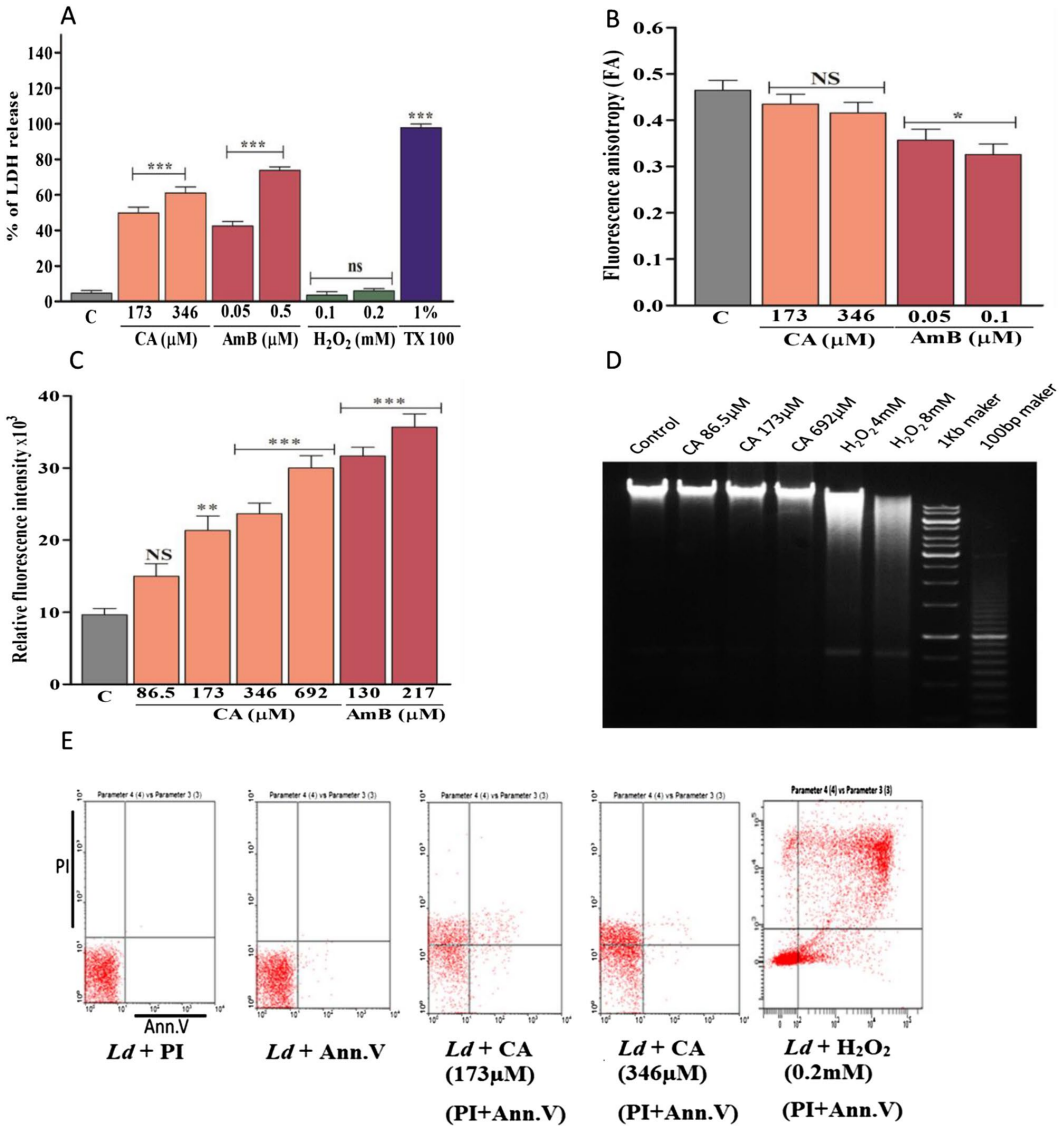
Measurement of membrane leakage by LDH assay after 6 h, 1% Triton-X-100- (Tx-100)-treated and H_2_O_2_-treated (0.1–0.2 mM) cells were used as positive and negative control respectively. CA and AmB-mediated membrane damage of promastigotes measured by LDH assay (**A**). Determination of fluorescence anisotropy was performed after caprylic acid treatment and values of DPH-labelled parasites were compared with AmB treated cells (**B**). Reactive oxygen species (ROS) is measured after 12 h treatment with CA (**C**). Agarose (1.5%) gel electrophoresis of genomic DNA isolated from parasites treated with CA and H_2_O_2_ with 100 bp and 1 kb DNA ladder as a marker (**D**). FACS analysis of AV and PI-stained promastigotes after treatment with CA (173 and 346 μM). Untreated cells were used as negative control and H_2_O_2_ (0.2 mM) treated cells as positive control of apoptosis (**E**). Note: **0.05 < P < 0.01; ***0.01 < P < 0.001.

Lactate dehydrogenase (LDH) assay is the indicator of membrane damage and associated necrosis caused by antileishmanial agents^24,25^. After 6 h of treatment at 2× IC_50_ (346 µM) concentrations, LDH release was ~ 27% (P < 0.001) and ~ 38% (P < 0.001) for CA and AmB-treated promastigotes, respectively (Fig. 3A). After 12 h, under similar treatment conditions, the LDH release was ~ 61% and ~ 73% for CA and AmB, respectively. Promastigotes treated with H_2_O_2_ and Triton-X-100 were used as negative and positive controls, respectively.

The decreased fluorescence anisotropy which is an indicator of increased membrane fluidity due to membrane damage is measured by fluorescent probe diphenyl-hexatriene (DPH) using AmB as a control^24^. The fluorescence anisotropy value of CA-treated (346 µM) promastigotes was lower than AmB treated cells and was found to be unchanged with increasing dose (Fig. 3B). Therefore, membrane fluidity is lower in CA-treated cells than in AmB-treated cells, indicating less membrane damage.

### Apoptosis is not observed in CA-treated parasites

Since membrane damage and LDH release were observed for CA-treated cells; ROS measurement, DNA fragmentation, and FACS analysis was done for the evaluation of necrotic or apoptotic mode of cell death. With an increasing dose of CA, ROS found to be increased (Fig. 3C) in parasites. However, there was no DNA fragmentation indicative of apoptosis in CA-treated cells although H_2_O_2_-treated cells show significant DNA fragmentation (Fig. 3D). Further FACS analysis shows increased propidium-iodide-positive (PI+) cells than Annexin-V-positive (AV+) cells with an increasing dose of CA indicating more necrosis than apoptosis (Fig. 3E). In case of H_2_O_2_-treated (0.2 mM for 6 h) LD cells, used as apoptotic control, AV+ late apoptotic cells were observed (~ 21%) as expected (Fig. 3E).

### Ergosterol depletion is a major reason for death which is reversed by ergosterol supplementation

**Figure 4:**
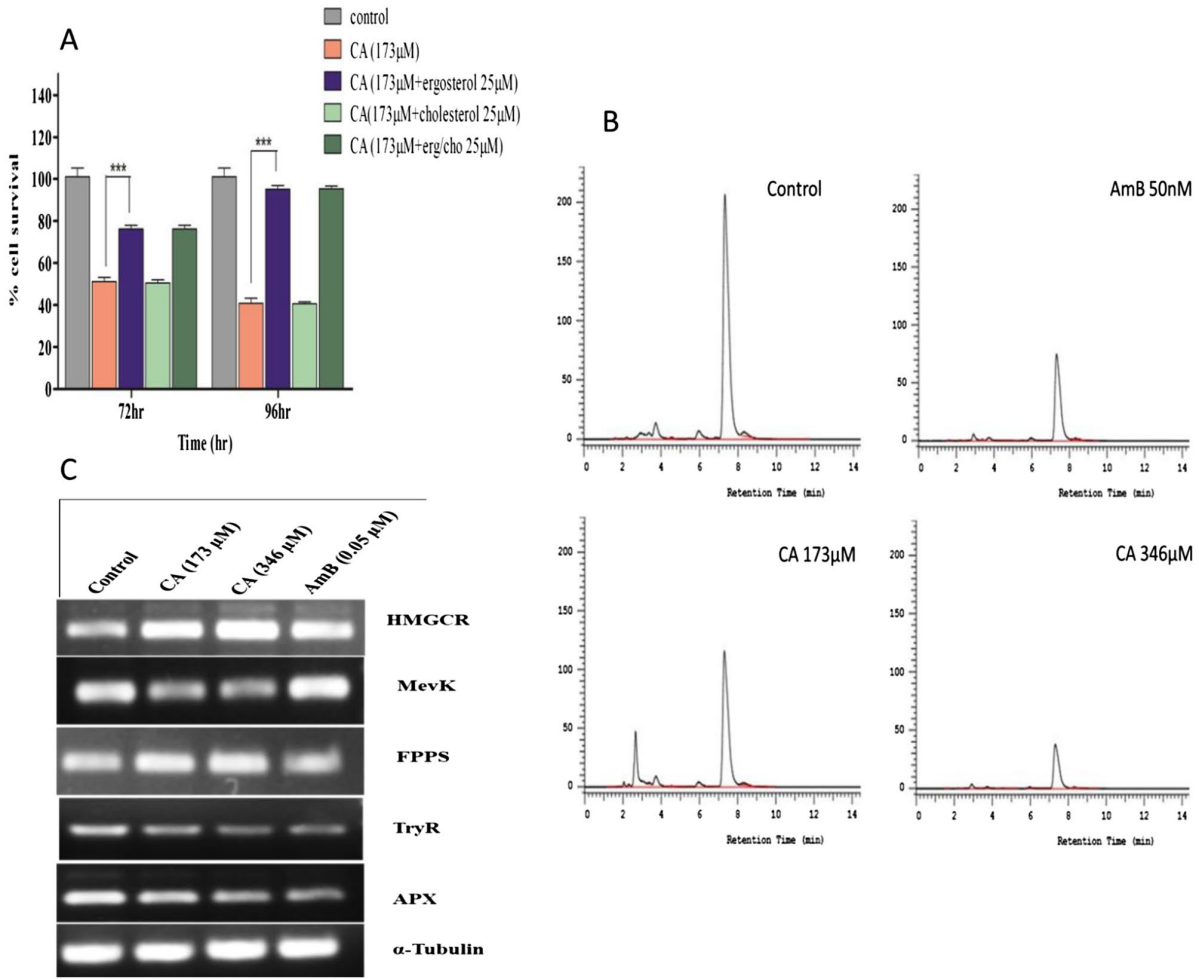
Sterol supplementation study along with cell viability by pretreating the promastigotes with ergosterol/cholesterol followed CA treatment at different time intervals (**A**). Measurement of ergosterol content in untreated parasites or treated with caprylic acid and AmB (**B**). Gene expression profiling was studied by semi-quantitative PCR of parasites after treatment with CA and AmB for 12 h (**C**). Here α-tubulin was used as an internal control.

Ergosterol (ERG) is a key component of the LD cell membrane along with cholesterol. AmB kills the parasite by binding with ERG with higher efficacy than cholesterol (CHO)^25^. We reasoned that membrane damage in CA-treated cells, as measured by increased LDH release, may be associated with sterol depletion. The viability of CA-treated (173 µM) cells was measured with or without pre-treatment with 25 µM of ERG, CHO, and ERG + CHO for 72 and 96 h. Pre-treatment of CA-treated parasites changes cell survival from ~ 51 to ~ 76%, ~ 51%, and ~ 77% for ERG, CHO, and ERG + CHO, respectively after 72 h (Fig. 4A, P < 0.001). Therefore, survival of CA-treated LD cells are only observed when they are pretreated with ERG. Similarly after 96 h the cell survival changes from ~ 40 to ~ 95%, ~ 40%, and ~ 96% due to pre-treatment with ERG, CHO, and ERG + CHO, respectively. Therefore, ERG supplementation revives cell growth significantly during CA treatment indicating depletion of ERG as a major cause of death. So, the ERG content of promastigotes was measured based on the ERG standard curve with a retention time of 7.31 min in HPLC^24^. The ERG content was ~ 32% (267.6 vs. 180.7 µg/ml) and ~ 66% (267.6 vs. 90 µg/ml) reduced compared to untreated cells with 173 µM and 346 µM dose of CA after 12 h of treatment (Fig. 4B). For 173 µM dose of AmB treatment, the ERG content was reduced to ~ 44% (267.6 vs. 150.8 µg/ml) after 12 h. Therefore, the antileishmanial effect of CA is associated with depletion of ERG and can be reversed by ERG supplementation.

### Gene expression studies show decrease in mevalonate kinase expression level

Semi-quantitative PCR was done for the genes HMG CoA Reductase (HMGCR), MevK, and Farnesyl pyrophosphate synthase (FPPS) from the RNA of LD cell after treatment with 173 µM and 346 µM doses of CA. AmB and untreated cells were used as the positive and negative control, respectively. HMGCR and FPPS showed ~ 2, ~ 2.3, and ~ 1.5, ~ 1.65-fold higher expression in 173 μM and 346 μM doses of CA, respectively compared to untreated control (Fig. 4C). AmB treated cells showed ~ 1.3 foldchange in HMGCR but no changes in FPPS expression level. For MevK, under similar conditions, the expression level was reduced by ~ 1.8 and ~ 2.1-fold, respectively, while the AmB treatment had no effect (Fig. 4C). HMGCR is known to be the rate-limiting enzyme in the ERG biosynthesis pathway^26^ and therefore its change in expression level provides a strong indication that the ERG biosynthesis pathway is the target of CA. The decrease in MevK expression in CA-treated cells allows accumulation of upstream metabolites which is, therefore, associated with an increase in HMGCR expression. The expression level of ascorbate peroxidise (APx) and trypanothione reductase (TryR), the enzymes responsible for maintaining the oxidation–reduction homeostasis in LD, showed no significant change under similar conditions. The expression level of the α-tubulin gene, used as a loading control, was found to be unchanged under different treatment conditions.

### Docking and MD simulation studies identified MevK as a possible target for caprylic acid

**Figure 5:**
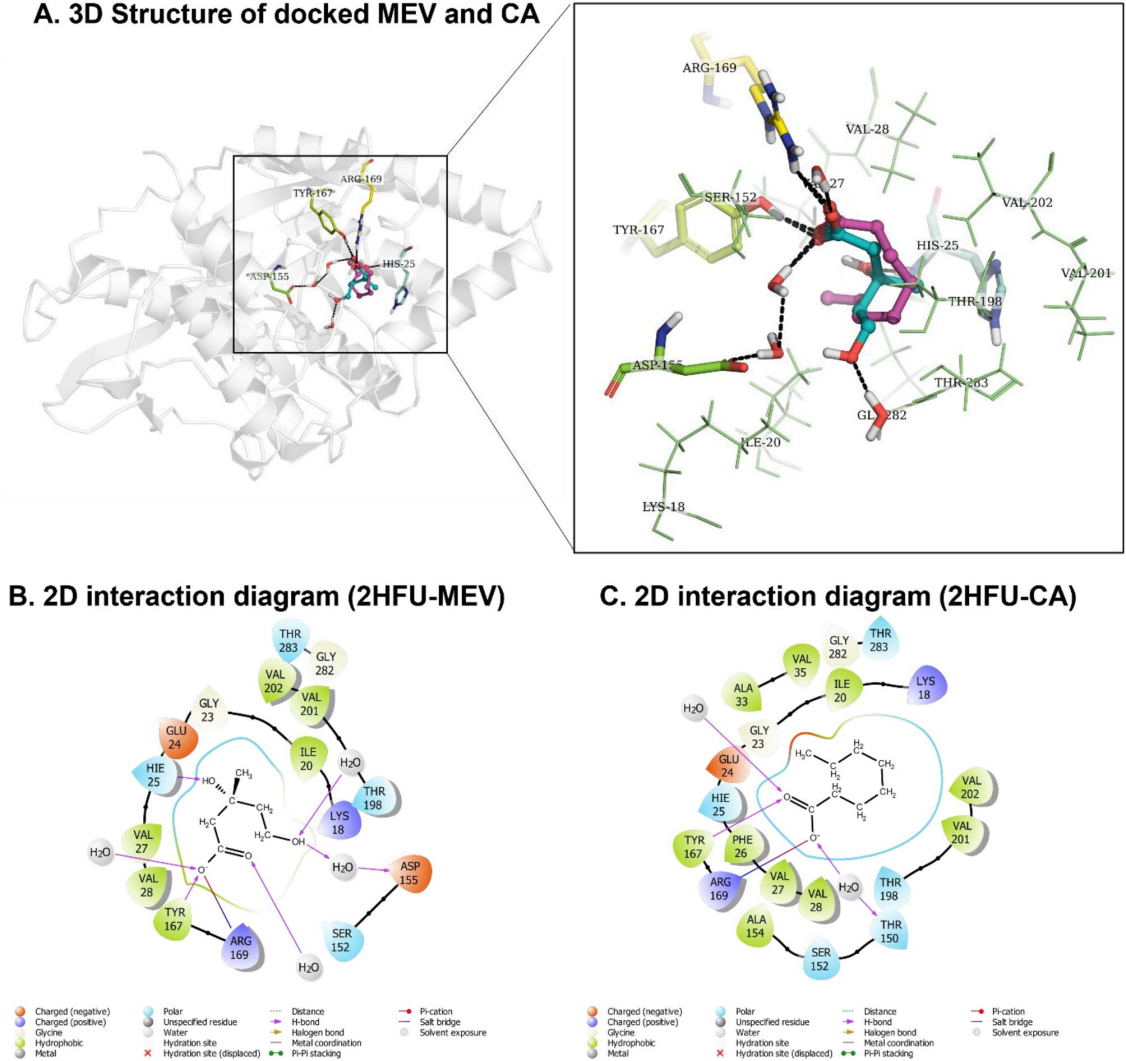
(**A**) 3D structure of *L. major* mevalonate kinase in complex with docked R-mevalonate (MEV: cyan color) and caprilic acid (CA: purple color). Ligand is shown in ball and stick representation, binding site residues shown in line (non H-bonds interactions residues) and thick sticks (H-bonds interaction residues) representation. Water molecules were shown in red color (thin sticks representation). H-bonds interaction shown via dashed black line. (**B**,**C**) Shows 2D interaction diagram of MEV and CA, respectively. Color scheme used in figure is based on types of interactions, shown in figure.

**Figure 6:**
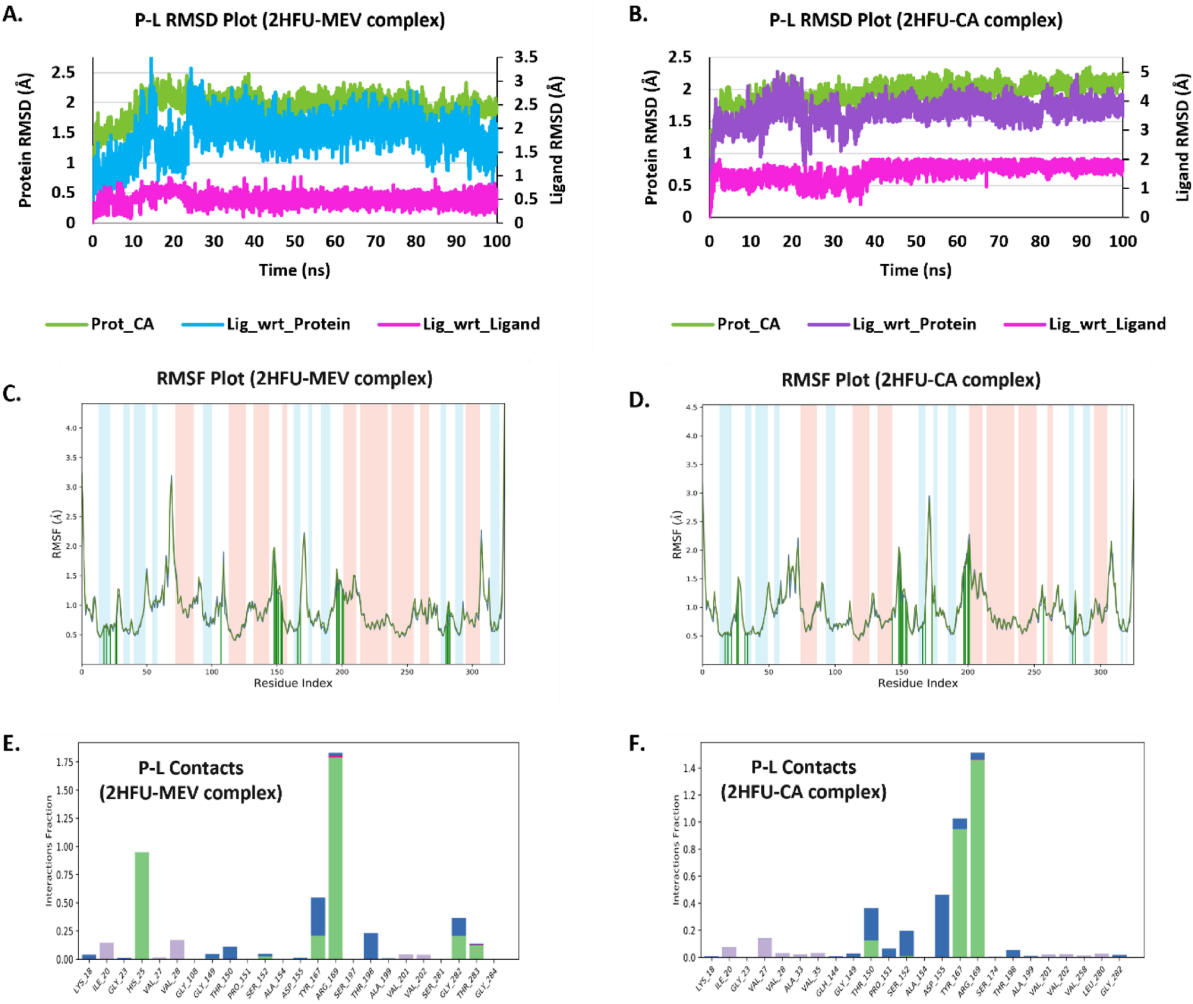
Shows the MD simulation trajectories analysis of both Mevalonate kinase complexed with R-Mevalonate (MEV) and Caprylic acid (CA). (**A**,**B**) Shows the RMSD result of a protein (left Y-axis) and ligand (right Y-axis). The RMSD of a ligand is calculated by aligning the protein–ligand complex on the reference protein backbone and then measuring the RMSD of the ligand heavy atoms (Lig_wrt_protein). If the observed values are significantly greater than the protein's RMSD, the ligand has most likely diffused away from its original binding site. The RMSD of a ligand aligned and measured only on its reference conformation is shown in 'Lig fit Lig.' Internal ligand atom fluctuations are measured by this RMSD value. (**C**,**D**) The RMSF plot depicts the distribution of alpha helixes (red colour) and beta sheets (blue colour) as well as local fluctuations for a protein chain throughout the simulation. The ligand contacts points with specific residues are shown in green bars. (**E**,**F**) The interaction fraction of individual protein residues with ligand via H-bonds (green), Salt bridge (blue), hydrophobic (purple), and ionic (pink) interactions is shown in the protein–ligand contacts plot. Due to the involvement of a particular residue in multiple types of interactions, the total value of interaction fraction may be greater than 1.

Based on the ERG supplementation assay and gene expression analysis we reasoned MevK and/or the other enzyme(s) of the ERG biosynthesis pathway of LD may be the molecular target of CA. We observed that mevalonate (3,5-Dihydroxy-3-methylpentanoic acid), which is converted by MevK to mevalonate 5 phosphate, has a close structural similarity with CA. The available crystal structure of *L. major* MevK with R-mevalonate (PDB ID 2HFU) provides a suitable template for docking studies using R-mevalonate and CA as substrate and inhibitor, respectively^27^. This is because the MevK of *L. major* and *L. donovani* have 96.3% structural identity. CA shows almost similar orientation and interaction with the residues in the binding site of MevK with respect to R-mevalonate. The CA and R-mevalonate show almost comparable binding energy of − 7.489 and − 8.991 kcal/mol with the MevK enzyme respectively in docking studies. To further investigate the stable interaction of CA and R-mevalonate with the binding site of MevK, we performed a 100 ns MD simulation. The root means square deviation (RMSD) of MevK-CA and MevK-R-mevalonate complex has been stabilized after about 10 ns (Fig. 6A,B). The root mean square fluctuation (RMSF) plot indicates that amino acids encompassing the residues 150–200 of N-terminus of MevK including the residues Tyr167, Arg169 and adjacent amino acids show similar molecular motion when bound to mevalonate and CA, respectively (Fig. 6C,D). The MD simulations revealed that carboxyl oxygen of CA interacts with the side-chain of Tyr167 and Arg169 via H-bond (Fig. 5A), a flexible and conserved region between β5 and β6 found in some species. Moreover, one water molecule stabilizes the orientation of CA by forming a bridge between the carboxyl oxygen of CA and the side chain of Thr150. While R-Mevalonate carboxyl oxygen interacts with the side-chain of Tyr167 and Arg169 (Fig. 5B). Furthermore, one of the hydroxyl groups of R-Mevalonate forms an H-bond with the His25, a key residue responsible for stereoselective interaction with R/S-mevalonate, while the terminal hydroxyl group interacts with the Thr198 and Asp155 through water bridges. Therefore, a strong H-bonding network of CA and R-Mevalonate with Tyr167 and Arg169 in the binding site of MevK is crucial for binding. Whereas H-bonding through bridged water molecule further stabilizes CA in the binding site of MevK (Figs. 5B,C, 6E). The weak hydrophobic interaction of Ile20, Val27, and Val28 with CA, indicates the stabilized conformation of the ligand, and it is superimposing to the binding of R-mevalonate (Fig. 6C,F).

### Recombinant LD-MevK was purified for enzyme assay with drug target validation

**Figure 7:**
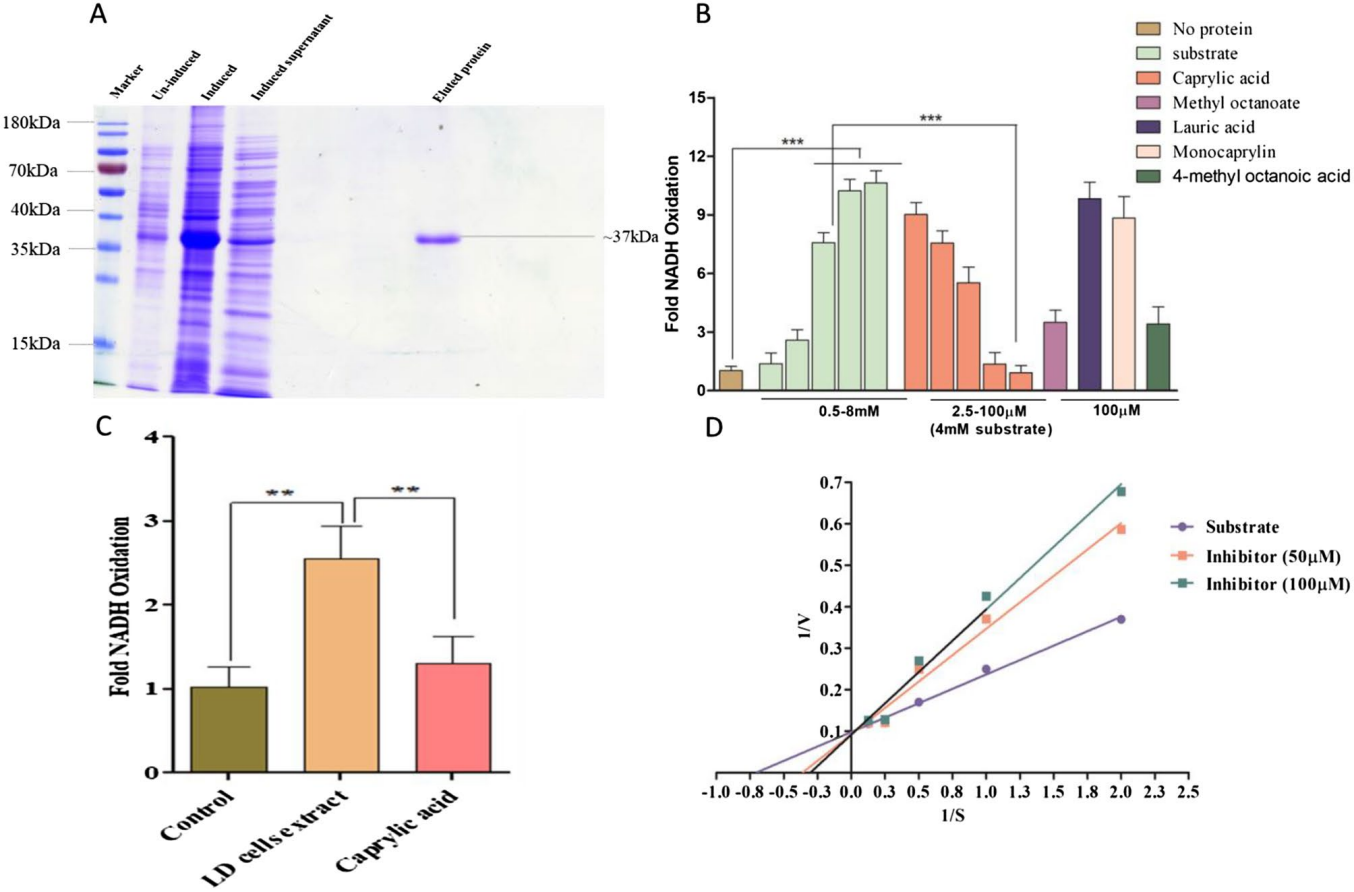
10% SDS-PAGE analysis of purified LD-MevK (**A**). MevK enzyme activity was measured as fold change in NADH oxidation, with CA and its analogues (**B**). Enzyme activity *L. donovani* cell extract in presence and absence of CA (**C**). LB plot of enzyme kinetics assay indicating K_m_ and V_max_ (**D**). Note: **0.05 < P < 0.01; ***0.01 < P < 0.001.

To elucidate the role of MevK as a possible target we cloned, expressed, and purified recombinant LD-MevK from transformed *E. coli*. Further, the mutant Y167F protein was generated to check the efficacy of mutation in active site, compared to WT MevK, since Tyr167 was found to be an important amino acid in binding with the carboxylate group of CA by docking studies. PCR was used to amplify the MevK gene (Gene ID-13385869) using LD genomic DNA as a template. A distinct band of about 1 kb was observed (SI-3A) in 1% agarose gel. Purified PCR product was double digested with BamHI and HindIII restriction enzymes and cloned into the pET28a vector. The positive clones were selected by colony PCR and restriction enzyme digestion of isolated plasmids (SI-3B). Clones are further confirmed by sequencing. Protein expression was performed in BL-21 (DE3) cells using 0.1 mM IPTG as inducer at 37 °C. The over-expression of MevK was observed at ~ 37KDa in SDS-PAGE (SI-3C) which was further confirmed by Western blot using an ant-his antibody (SI-3D). The MevK protein was purified from a supernatant fraction of 100 ml of cultured bacterial cells using Ni-NTA agarose beads using standard procedures (Fig. 7A). The mutant Y167F was generated by site-directed mutagenesis using cloned WT-MevK pET28a vector as a template and the recombinant mutant MevK (Y167F) was purified similarly (SI-5).

### Caprylic acid and it’s analogs act as competitive inhibitors for LD-MevK

The pyruvate kinase–lactate dehydrogenase coupled assay system was used for the measurement of the activity of MevK where a decrease in absorbance of NADH at 340 nm is correlated with increased enzyme activity^28,29^. Recombinant MevK activity was represented as fold change in NADH oxidation^30^ in presence of increasing concentration (0.5–8 mM) of R-mevalonate (Fig. 7B). The purified enzyme shows maximum activity at pH 9.0 (Fig. SI-4B) and temperature of 37 °C (Fig. SI-4A). In presence of different divalent and monovalent salts, alone, at lower concentrations (5 mM) the activity of the enzyme was checked. The maximum enzyme activity in presence of different salts follows the order Mg^2+^ > Mn^2+^ > Ca^2+^ > Zn^2+^ > Cu^2+^ (Fig SI-4C). In presence of 5 mM of NaCl alone, the activity is reduced by ~ 87% (Fig. SI-4C) compared to the activity obtained with 5–25 mM MgCl_2_ alone. However, the presence of NaCl and MgCl_2_ in the mixture does not cause any inhibition in MevK activity. The presence of MgCl_2_ (5–25 mM) in the reaction mixture, irrespective of the presence of other divalent/monovalent ions always maintains the high enzyme activity. This indicates the presence of Mg^2+^ as a cofactor for MevK. Henceforth, all enzyme assays are done at pH 9.0 with 4 mM R-mevalonate and 5 mM MgCl_2_ at 37 °C. With the increase in substrate concentration from 0.5 to 4 mM the increase in MevK activity was from ~ 1.3 to ~ 10.2 fold. The value of K_m_ and V_max_ was 1.42 mM and 10.2 µmol/min, respectively for R-mevalonate. The activity of MevK was reduced ~ 41%, ~ 96%, and ~ 99% in presence of 25, 50, and 100 μM of CA, respectively. In presence of ~ 35 µM CA, the enzyme activity is reduced by 50%. In presence of 100 µM of LA and monocaprylin, there was no inhibition of MevK activity. However, in presence of 4m-CA and 1m-CAE, which maintains the C8 backbone, and either carboxyl or ester group at the terminal, the activity is reduced by ~ 65% (Fig. 7B). This is further corroborated by the docking studies with MevK (Fig. SI6). This confirms the essentiality of the presence of a linear C8 backbone in the inhibitor(s) for accommodation and binding to the active site of LD MevK and consequent inhibition of enzyme activity. The Lineweaver–Burk (LB) plot revealed that CA acts as a competitive inhibitor with nearly unchanged V_max_ (~ 10.2 µmol/min vs. ~ 10.8 µmol/min) but with increased K_m_ (~ 1.42 mM vs. ~ 2.76 µM) in absence and presence of 50 µM of CA (Fig. 7D). At 100 µM of CA, the K_m_ value increase to 3.29 mM and V_max_ is 10.90 µmol/min (Fig. 7D). Interestingly, the enzyme activity of mutant Y167F MevK was found to be reduced ~ 55% compared to WT MevK. This indicates the importance of Tyr167 residue in the active site of MevK for binding with R-mevalonate as predicted by docking studies.

### Homologous overexpression of MevK in parasites resist caprylic acid-mediated killing

**Figure 8:**
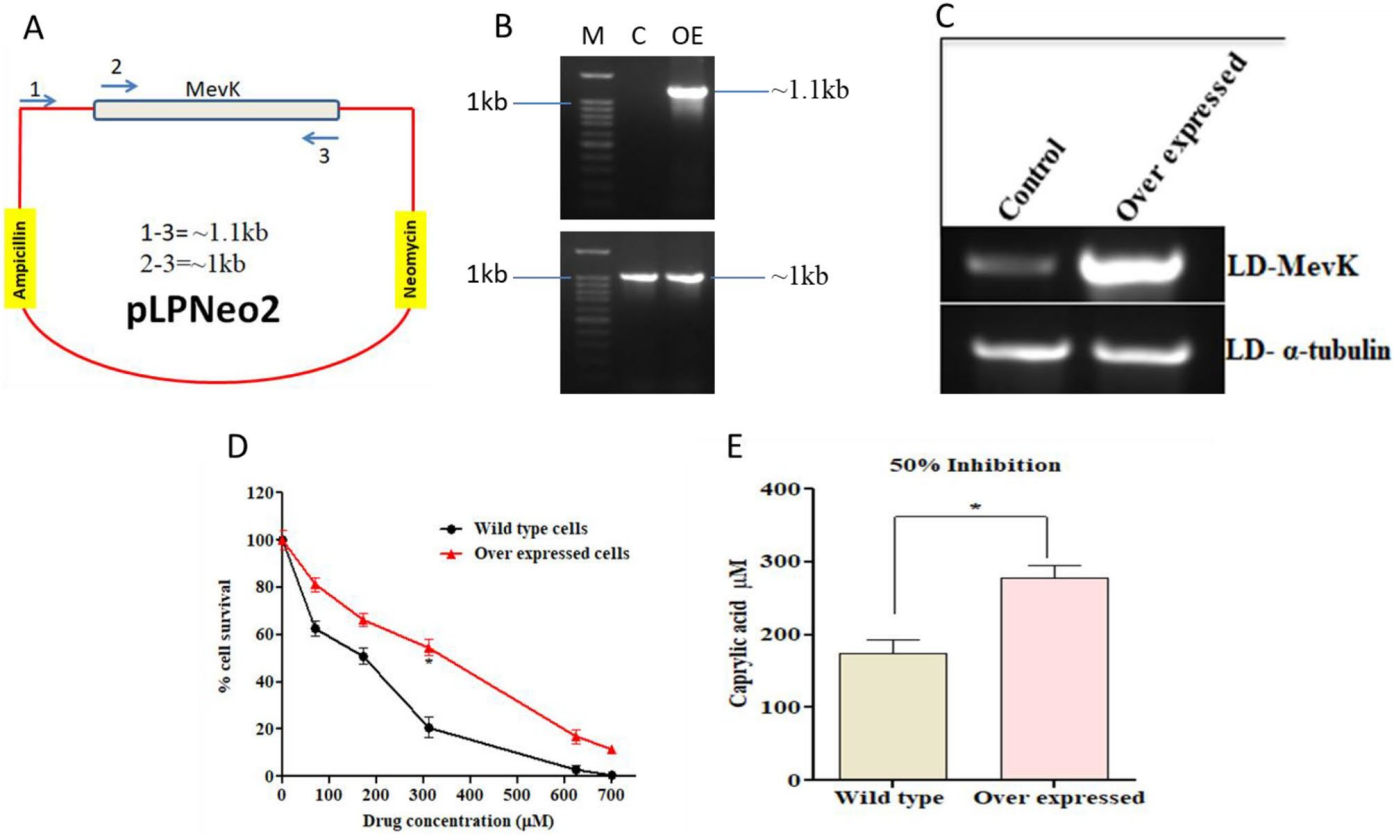
Evaluation of MevK overexpression in *L. donovani* after cloning in pLpNeo2 expression vector primers used for confirmation of MevK expression from pLpNeo2 in LD is shown by diagram (**A**). PCR from transfected and non-transfected cells by primer pair 1–3 and 1–2 (**C**). Further, semi-quantitative PCR was done to confirm the overexpression (OE) of MevK gene whereα-tubulin was used as an internal control (C). Cell survival with IC_50_ concentration was measured on transfected and non-transfected cells after treatment with CA (**D**,**E**). Note: *P < 0.05.

The role of CA in MevK inhibition was further confirmed by activity assay with LD cell lysates after homologous expression of MevK using pLpNeo2 expression vector^31^. The maintenance of the expression vector in LD was done by selecting the transfected cells in 100 µg/ml of neomycin (Fig. 8A) and confirmation of a PCR product of ~ 1.15 kb (Fig. 8B). The PCR product of ~ 1 kb was observed from both, transfected and non-transfected, LD cells since the primer pairs 2 and 3 amplify the MevK gene from the chromosome irrespective of the presence/absence of transfected pLpNeo2-MevK in LD cells (Fig. 8A). However, the primer pair 1 and 3 can only give amplification from the cloned pLpNeo2-MevK vector since the forward primer 1 is specific for vector only. We confirmed ~ 3.2 fold over-expression of MevK mRNA using semi-quantitative PCR (Fig. 8C). Consequently, the MevK activity is increased by ~ 2.5 fold in the LD cell lysate of pLpNeo2-transfected cells but subsequently decreased by ~ 48% in presence of 100 µM of CA (Fig. 7C). Also, LD cells containing over-expressed MevK show more resistance against the dose-dependent killing by CA compared to non-transfected LD cells with an effective increase of IC_50_ of ~ 1.6 fold (Fig. 8D,E). All these results, established, further, the role of MevK as a molecular target for CA in LD.

### ITC studies confirm strong binding interactions between MevK and CA

**Figure 9:**
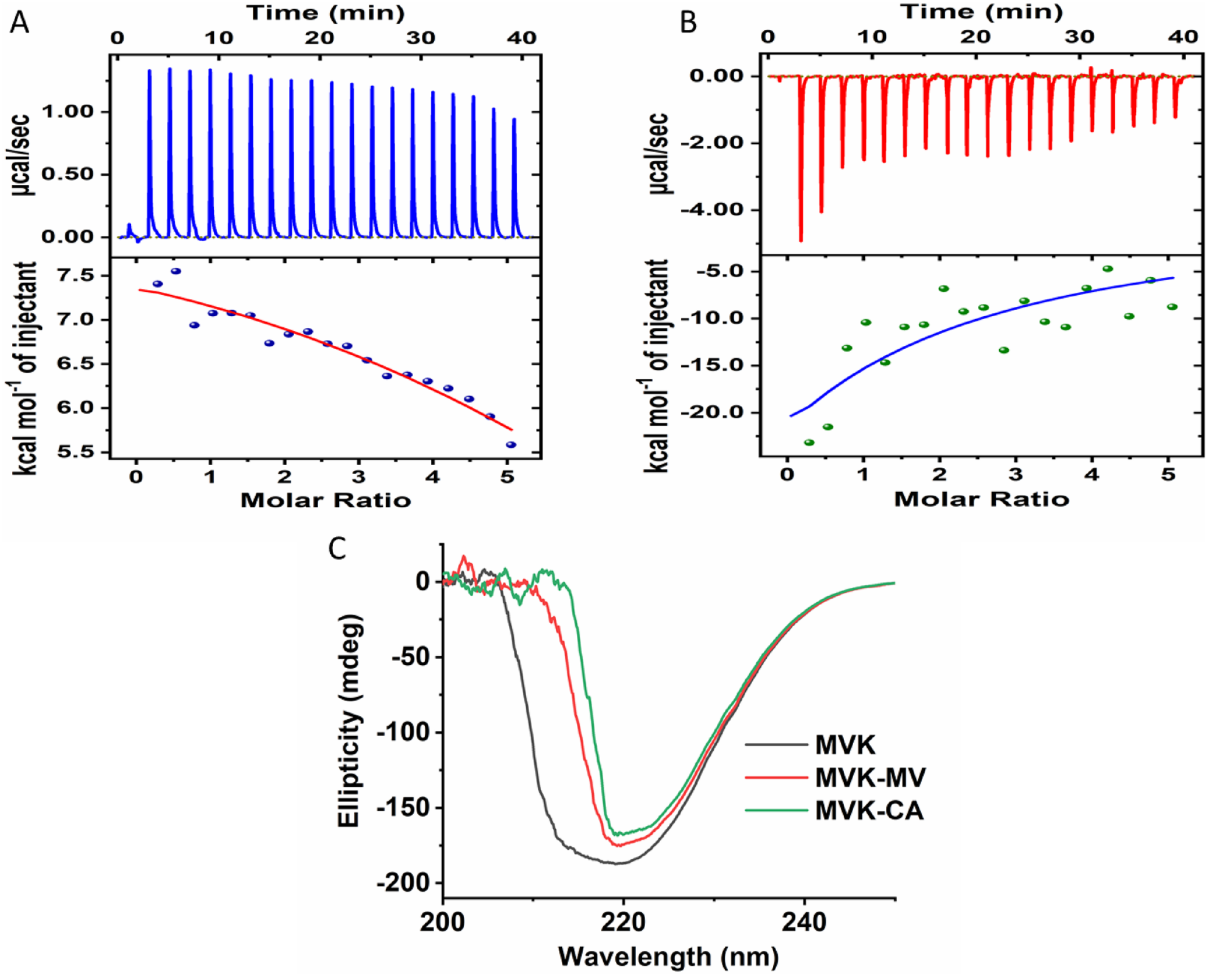
Isothermal calorimetry (ITC) was performed to confirm the binding of CA (**B**) or R-mevalonate (**A**) with LDMevK enzyme. CD spectra of MevK, MevK with substrate (R-mevalonate) and MevK with inhibitor (CA) was analyzed (**C**).

The high affinity of CA towards MevK was further corroborated by ITC studies. The ITC isotherm of MevK-CA showed that the binding interaction of CA with MevK is exothermic with a binding constant of (9.90 ± 3.47) × 10^3^ M^−1^ (Fig. 9B). Whilst, the ITC isotherm of MevK-R-mevalonate showed that the binding interaction of R-mevalonate with MevK is endothermic with a binding constant of (1.59 ± 1.94) × 10^4^ M^−1^ (Fig. 9A) which is about two times higher than that of CA. But most importantly, the binding of CA is thermodynamically favored whereas the binding of R-mevalonate is thermodynamically unfavoured further confirming the potential of CA to be an excellent competitive inhibitor to LD-MevK.

### Circular dichroism studies confirm similar conformational changes of MevK after binding with substrate and CA

Conformational changes of MevK in the presence of CA were analyzed through circular dichroism (CD) studies. A drastic change in spectra was observed when CA was mixed with MevK. The negative peak around 214 nm completely vanished and the peak intensity at 220 nm was reduced significantly indicating a decrease in α-helicity and an increase in the β-sheet structure of MevK. CD spectra of MevK-R-mevalonate were used as a control. Notably, the CD analysis reappraises the high affinity of CA for LD-MevK (Fig. 9C).

### Proteomics analysis by LC-HRMS with CA-treated LD promastigotes showed high downregulation of MevK

**Figure 10:**
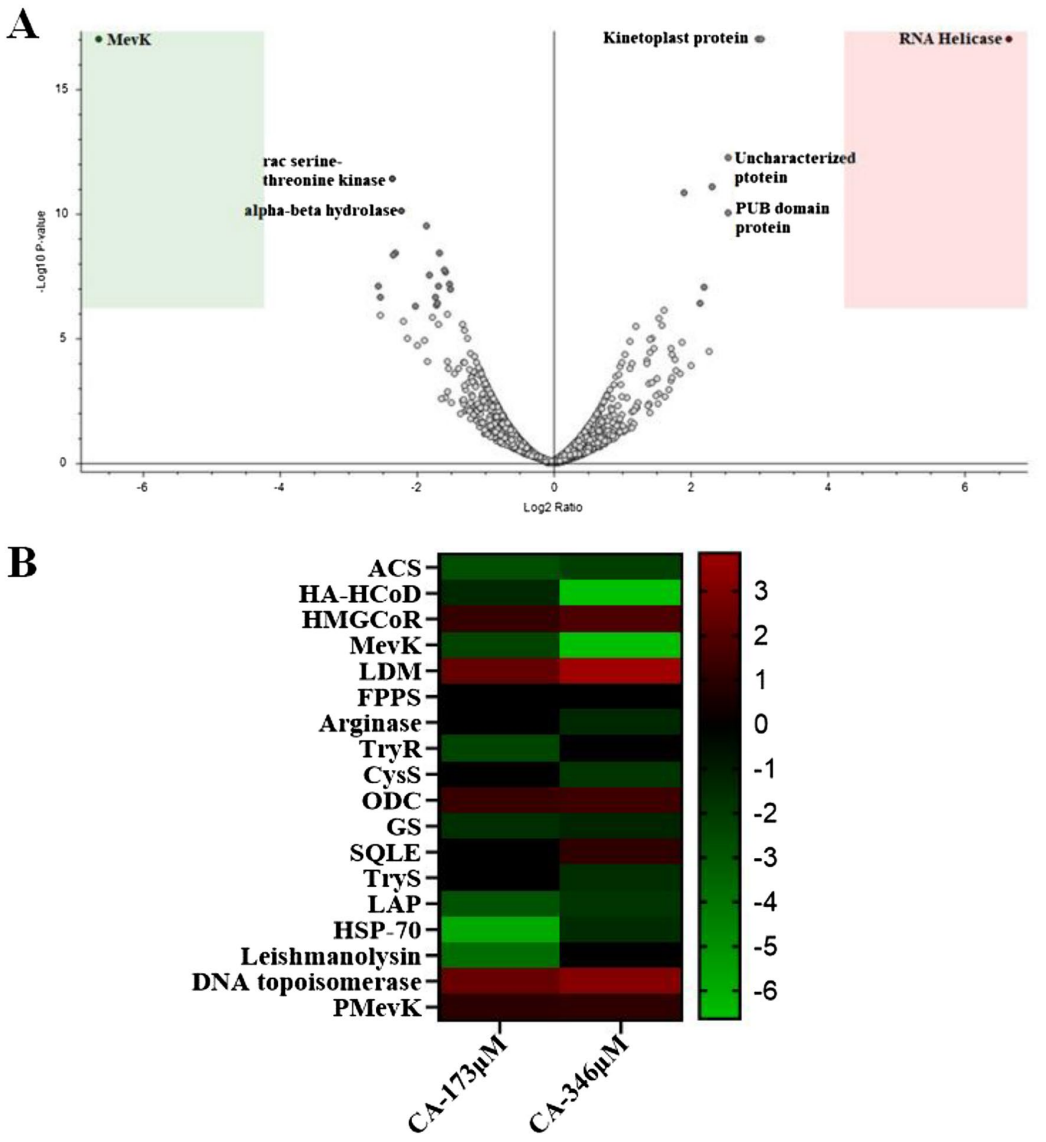
Principal component analysis (PCA) plot of protein expressions (LC-HRMS; Proteome discoverer database). An enzyme on the left side is up-regulated and on the right side, it is down-regulated. Fold change in protein expression level after treatment with 346 µM CA compared to untreated control. Red and green color indicates upregulation and downregulation of proteins, respectively after CA-treatment compared to untreated cells with the fold change indicated at the right.

The change in the expression level of various proteins involving different metabolic pathways was analyzed by proteomics analysis of CA-treated promastigotes in presence of two different doses of CA (173 and 346 µM). The volcano plot (Fig. 10) shows the number of proteins expressed as down-regulated or up-regulated in two arms of the plot with fold change in expression after treatment with 346 µM CA compared to untreated cells. Proteins showing > 1.5 to 2-fold change in expression and having a consistent trend of either down-regulation or up-regulation in both doses of CA were considered significant. In Fig. 10B, we have used a heat map where green and red color is used to show the down- and up-regulation of different proteins for CA treated parasites compared to untreated control.

Change in protein expression levels in the ERG biosynthesis pathway was found to be significant in CA-treated cells compared to untreated control. Acetyl-coenzyme A synthetase which is the enzyme responsible for the synthesis of acetyl CoA from acetate^32^ and supplies the precursors for the FA and ERG biosynthesis pathway is down-regulated in both CA-treated samples by 2.7 and 2.2 fold, respectively. The HMGCR was upregulated 1.25 and 1.8 fold with the treatment of 173 and 346 µM, respectively. This is possibly a consequence of the high down-regulation of MevK enzyme, which acts downstream of HMGCR in the ERG biosynthesis pathway. The analysis confirmed that there is a significant decrease in MevK protein expression level (~ 2.3 fold with 173 µM CA) and (~ 6.6 fold with ~ 346 µM CA) compared to untreated control (SI-Table 2). That high level of decrease in the MevK expression confirms, further, the role of MevK inhibition by CA as the major cause of parasite death. However, expression of other enzymes of ERG biosynthesis which are downstream of MevK pathway (SI-7) e.g., phosphomevalonate linase, squalene epoxidase and lanosterol 14α-demethylase (LDM) are almost unchanged (Fig. 10B and SI-Table 2). cysteine synthase (CysS), glutathione synthase (GS), and trypanothione reductase (TryR)—the enzymes which are involved in the redox biosynthesis pathway and important for LD survival are also down-regulated ~ 1.8, ~ 1.6, and ~ 2.4 respectively in CA-treated cells (Fig. 10) consistent with increased ROS production (Fig. 3C). Apart from inhibition of acetyl-CoA synthetase (ACS) which is necessary for supplying the precursor for the FA biosynthesis pathway, the enzyme (3R)-3-hydroxyacyl-CoA dehydratase (HA-HCoD), a key enzyme of lipid metabolism was also found to be down-regulated significantly (~ 1.4 and ~ 6.6 fold) under similar conditions. Leishmanolysin, a membrane-bound surface glycoprotein and one of the major antigens involved in host infections is ~ 3.8 fold down-regulated after CA treatment. Heat shock protein 70 (HSP-70), involved in stress response and leucyl aminopeptidase (LAP), involved in free amino acid regulation are important therapeutic targets in LD. These proteins are downregulated by ~ 5.9 and ~ 2.9 fold respectively, after treatment with 1 × dose of CA compared to untreated control. We found upregulation of DNA topoisomerase, an important drug target in LD, by ~ 2.6 and ~ 3.3 fold in presence of two different doses of CA. However, how this upregulation is linked to CA-mediated parasite death needs further studies. No significant change in expression of others proteins of ERG biosynthesis pathway and polyamine biosynthesis proteins were observed (SI-7). Thus, proteomics analysis confirms the down-regulation of MevK protein as one of the major molecular mechanism of CA-mediated death of LD.

## Discussion

Historically, raw materials and compounds isolated from natural resources were used as the main sources of medicine to treat a wide spectrum of human diseases^33^. Between 1983 and 1994, 39% of the 520 new approved drugs were natural products or their derivatives, and 40–80% of antibacterial and anticancer drugs were from natural origins^34^. Due to the advancement of chemical synthesis, high throughput screening, and fermentation technology drug development from natural resources had decreased in the current decades. However, between 1999 and 2013, FDA had approved 28% of drugs which were developed either based on natural pharmacophores or their minor structural modifications^35^. Natural product-based drug delivery is possibly most important for NTDs where there is a constant lack of interest and funding from private partners due to low benefits in revenue^6^. These NTDs, comprised mostly of leishmaniasis and Chagas disease, affect > 875 million children in developing economies along with 1 billion people annually with a death rate of 500,000 per year. Lack of vaccine and emergence of toxicity and drug resistance against all mainline chemotherapeutics against leishmaniasis attracts drug developments strategies with natural resources^36^. The biosynthesis pathway of FA as a possible drug target has recently gained interest for leishmania^37^ although FA and its derivatives were well known to be antifungal, antimalarial, and antibacterial agents^38^. The fatty acids, CA and LA were not generally part of biological membranes and, therefore, evaluated as antibacterial or antifungal agents although the detailed molecular mechanism was not known. In this study, we wanted to explore the antileishmanial effect of different medium-chain FAs along with CA and their analogs against *L. donovani*, the causative agent for VL The IC_50_ of CA (~ 372 µM) against parasite-infected amastigotes was higher compared to any standard chemotherapeutic agents but the cytotoxicity of CA is very less (Figs. 1B and 2A). Even with 28 mM of CA, which is > 75 times of IC_50_, only 47% of human cells are killed. Since the efficacy decreased with increasing chain length, we reasoned that the C8 backbone and presence of a carboxyl/ester group is essential for killing. Commercially available analogs of C8 FAs (4m-CA, 1m-CAE, valproic acid, and 8-amino CA) were tested along with CA for antileishmanial efficacy. Interestingly, 4m-CA and 1m-CAE, which maintain the linear C8 backbone shows almost equal efficacy to CA (Fig. 1C). However, valproic acid (carrying C8 but branched side chains) and 8-amino CA (carrying a charged terminal –NH_2_ and –COOH group along with linear C8 backbone) lost complete activity against LD under similar conditions. This confirms the importance of the linear C8 backbone along with the terminal carboxyl/ester group for efficacy. LD cells were maintained at pH 7.5 and, therefore, CA having p*K*a of 4.9 will contain > 300 fold more of non-ionized than ionized carboxyl group in assay conditions. Since efficacy is mostly associated with the uncharged CA, compounds with –COOH (CA, 4m-CA) and –COOCH_3_ (1m-CAE) had a similar effect on LD cells. The non-ionized CA will also be more membrane-damaging than ionized CA which was confirmed by LDH and fluorescence anisotropy studies and correlates well with earlier studies on bacteria and yeast^13,20^. Although LDH release was significant the change in membrane fluidity was not found significant. We were unable to do the antileishmanial assay at pH < 6.5 since the parasite becomes non-motile and dies within 12 h. The amount of increased ROS production was dose-dependent and almost comparable to AmB-treated cells but there was no DNA degradation or apoptosis with indicated doses of CA. This proves that the primary mode of CA-mediated killing of parasites does not involve apoptosis but membrane damage may have an effect. Since LDH release was significant, we reasoned that ERG depletion leading to membrane damage may be important for parasite killing. ERG supplementation assay (Fig. 4A) which reverses the parasite killing and ~ 3-fold depletion of ERG content in 2 × IC_50_ doses of CA (Fig. 4B) confirms that depletion of ERG is the major reason for parasite killing. Docking studies showed that CA binds with LD-MevK with similar orientation and comparable binding energy to that of R-mevalonate. Interaction of Tyr 167 and Arg 169 is conserved for both, R-mevalonate and CA. Linear C8 backbone allows CA to fit in the active site with ionic interactions involving the –COOH group. This explains why 4m-CA, 1m-CAE shows similar antileishmanial efficacy by binding with MevK but not by 8-amino CA or valproic acid. MD simulation with stabilization of protein–CA complex within 10 ns suggests a strong interaction. ERG depletion, gene expression, and bioinformatics analysis by docking and MD simulation studies indicate that Ld-MevK is a target of CA. Recent studies with MevK knock-down LD cells have shown a ~ 60% decrease in ERG content^39^. This work also suggests that MevK has an important role in protecting the parasite from oxidative stress. The presence of MevK in the secretome of LD^40^, its direct association with host cells for enhanced phagocytosis and immune response leading to increased parasite survival suggests that inhibition of MevK could be a potential antileishmanial strategy^41^. We cloned and purified recombinant MevK for enzyme assays with CA as a possible inhibitor. The purified protein showed activity with increasing concentrations of R-mevalonate (0.5–8 mM) with maximum activity at alkaline pH (pH 9–10) and a temperature range of 25–37 °C. The activity of MevK was completely inhibited by 100 µM of CA but unaffected by LA (C12) and monocaprylin (monoglyceride of CA). Also, 4m-CA and 1m-CAE showed ~ 65% inhibition of MevK activity at 100 µM concentration. This suggests the necessity of a C8 linear backbone with a carboxyl/ester moiety for strong binding against MevK. The binding affinity as measured by ITC showed only a two-fold reduced affinity for CA than R-mevalonate against MevK. This strong binding of CA is also confirmed by the exothermic nature of MevK–CA interaction. Further, CD spectra of MevK-CA vs. MevK-R-mevalonate showed similar changes in MevK conformation, reaffirming the same binding site of CA and R-mevalonate on MevK. Proteomics analysis showed a specific trend of decreased down-regulation of MevK and increased up-regulation of HMGCoR with two different doses of CA (173 and 346 µM). This data explains, further, that CA binds with MevK specifically in a dose-dependent manner to down-regulate the protein expression of MevK which allows over-expression of the enzyme HMGCoR, present upstream of the pathway, indirectly. The enzyme acetyl-CoA synthetase which synthesizes acetyl CoA required for FA biosynthesis is also down-regulated with both doses of CA. This indicates a possible down-regulation of FA biosynthesis in CA-treated cells. In summary, we have established the molecular mechanism of CA-mediated cell death in leishmania involving the enzyme MevK as the primary target.

AmBisome is still the first line of treatment for VL but due to the high cost of lipid formulation, a low-cost alternative is always in demand^42^. Generation of new AmB derivatives^43,44^ or its nano-formulation with reduced toxicity^24,45–47^ was reported with some success. But due to the lack of vaccine^48^ and drug resistance and toxicity associated with AmB, the idea of nano and micro-formulation^49^, natural product-based drug-delivery^6^ using flavonoids or its formulation^50,51^, marine products^52^ and even drug-repurposing^53^ have gained significant attention for the treatment of various forms of leishmaniasis. In this study, we have confirmed the antileishmanial efficacy of CA, a natural FA that is already known to have antibacterial/antifungal efficacy. However, our data provide direct insight into the mechanism of action of CA against leishmania targeting the ERG biosynthesis pathway and involving the enzyme MevK. Disruption of membrane along with depletion of ERG by CA is associated with parasite death. To date, the major chemotherapeutic agents that have been identified as antileishmanial agents have targeted either the ERG biosynthesis pathway (AmB, azoles) or polyamine-dependent trypanothione pathway (flavonoids, SAG, etc.) which are inherently unique to parasites and absent in human. However, only a few enzymes like sterol demthylases^54^, TryR^55^, DNA topoisomearse^56^, etc. were successfully identified as enzymatic targets against chemotherapeutics in leishmania although any kinetic parameters for these drug–target binding are not available. In this study, we have rationalized the involvement of MevK as a target for CA using cell-based studies and bioinformatics analysis. Further, using enzyme assays with recombinant LD-MevK followed by ITC and CD studies the strong binding interaction between MevK and CA was established. Proteomics analysis of CA-treated cells and increased resistance of MevK-overexpressed parasites against CA-induced death, further, confirms the role of MevK as a target for CA. Thus CA, being a GRAS reagent, provides an encouraging opportunity to create a formulation with improved delivery in human trials of VL since its antifungal formulation has already shown success^57^.

## Materials and methods

### Materials

M-199, RPMI-1640, yeast peptone dextrose medium, Giemsa dye, Trypan blue, MTT, 2′,7′-dichlorodihydrofluorescein diacetate dye, *N*-acetyl l-cysteine (NAC), R-mevalonate lithium salt, phosphoenolpyruvate, lactate dehydrogenase, pyruvate kinase, β-NADH, ATP, glycine, diphenyleneiodonium chloride, ammonium bicarbonate, urea, sodium chloride, magnesium chloride, manganese chloride, zinc chloride, calcium chloride, copper sulphate, proteinase K, ergosterol (ERG), diphenyl hexatriene (DPH), RNase A, TRIZOL, nitro blue tetrazolium, the lactate dehydrogenase (LDH) assay kit, caprylic acid, 8-amino caprylic acid, 4-methyl octanoic acid, methyl octanoate, valproic acid, the Apoptosis Detection Kit, Taq Polymerase for PCR, Anti-His primary antibody and IgG-HRP conjugated secondary antibody and all solvents were from Sigma-Aldrich Co. (St Louis, MO, USA). The QIAamp DNA Mini kit, RNeasy Mini Kit, High fidelity Taq DNA Polymerase, and Ni-NTA agarose were from Qiagen. from Thermo-Fisher. The cDNA synthesis kit was from Hoffmann-La Roche (Basel, Switzerland). Trypsin, iodoacetamide, and DTT, restriction enzymes like BamHI, HindIII, and XhoI were purchased from Thermo Fischer (Waltham, MA, United States). The RAW 264.1 and THP-1 cell line was obtained commercially from the National Cell Repository, NCCS, Pune.

### Cell viability assay against promastigotes and amastigotes

*L. donovani* (MHOM/IN/1983/AG83) promastigotes were cultured as described^58^. Cells (1 × 10^6^/ml) were treated with FAs (C8–C18) at a 5–1500 µM concentration range. All FAs are dissolved in 100–200 mM of stock in DMSO and then serially diluted in a growth medium and treated with cells for 3 days and cell viability is measured by trypan blue exclusion and MTT (3-(4,5-dimethylthiazol-2-yl)-2,5-diphenyltetrazolium bromide) reduction method after every 24 h using standard procedures. Derivatives of CA (4m-CA, 1m-CAE, 8-amino CA) and valproic acid were also used in the same way. IC_50_ of each molecule was determined as described^43^. For amastigote assays macrophages were maintained in RPMI-1640 medium, infected with promastigotes, and IC_50_ was determined as described^59^. Briefly, macrophages were infected with the parasite with a multiplicity of infection of 1:10 and cultured on a plate for 12 h. Non-infecting parasites were removed by washing and parasite-infected macrophages were treated with different concentrations of CA, CRA, and LA (70–1500 µM) for 48 h and 72 h. Slides were washed and added with fresh medium for incubation in a CO_2_ incubator for 12 h. Macrophages infected with parasites but not treated with FA or derivatives were used as control. After methanol fixation and Giemsa staining of the slides, amastigotes from 100 to 120 macrophage nuclei per well were counted, using an oil immersion objective of a light microscope (Eclipse TS100; Nikon Corporation, Tokyo, Japan).

### Cytotoxicity and hemolysis assays

Cytotoxicity was measured against human THP-1 cells by MTT assay. Briefly, THP-1 cells were cultured with RPMI-1640 medium in 6 well plates (2 × 10^6^/well) and treated with CA (7, 14, 28 mM) and AmB (70, 350, 700 nM) for 48 h and cell viability was measured by MTT assay^42^. Hemolysis assay was done as described^60^. The concentration of CA used was 0.5, 1.5, and 8 mM while AmB is 100 µM. Briefly, RBCs from 3 ml blood was separated by centrifugation using histopaque. RBC of 4 × 10^8^ cells/ml in PBS was incubated with the concentrations 0.5, 3 and 5 mM of CA. After 4 h of incubation at 37 °C cells are centrifuged at 1500*g* for 5 min and supernatant was analyzed at 560 nm for measurement of hemolysis.

### Ergosterol determination by HPLC

Promastigotes (1 × 10^6^/ml) were treated with CA (173 µM and 346 µM) for 12 h. Pellets of treated and untreated cells (1 × 10^7^) were homogenized in 0.5 ml of 2:1 chloroform/methanol mixture for 30 min and then ERG was extracted for HPLC analysis^24,61^. The isocratic mobile phase of methanol:water (95:5) with a flow rate of 1 ml/min was used for separation. The peak of ERG was determined at 282 nm using a UV detector. Concentrations of ERG (dissolved in mobile phase) used for generation of the standard curve were 6.25, 12.5, 25, 50, 100, and 200 μg/ml. AmB (55 nM) treated promastigotes was considered a positive control for ERG depletion and measurement.

### Measurement of membrane fluidity by fluorescence anisotropy

Increased membrane fluidity is measured by decreased fluorescence anisotropy using DPH fluorescence. CA (173 µM and 346 µM) and AmB (0.05 µM and 0.1 µM) were used to treat promastigotes (2 × 10^6^ cells/ml) for 12 h, respectively. Cells are washed and resuspended in PBS followed by treatment for 1 h at 37 °C with DPH (2 µM). At 365 nm, the membrane-bound DPH probe was excited and the intensity of emission was recorded at 430 nm using a spectrofluorometer (LS 55; PerkinElmer Inc.). The value of Fluorescence Anisotropy was computed using the formula FA = [(I_II_ − I_⊥_)/(I_II_ + 2I_⊥_)], where I_II_ and I_⊥_ are the fluorescence intensities aligned perpendicular and parallel to the stimulated light's polarisation direction, respectively. The anisotropy values were measured and plotted as described earlier^24,62^.

### Promastigote membrane leakage determined by LDH assay

The release of intracellular LDH, which is linked to cell membrane damage and necrosis is measured from CA (173 µM and 346 µM) and AmB (0.05 and 0.5 µM)-treated promastigotes (5 × 10^6^ cells/ml in PBS buffer) after 12 h of treatment. The assays were done as described^24,63^.

### DNA fragmentation assay

Parasites (1 × 10^6^ cells/ml) were incubated with CA (86.5, 173 and 692 µM) for 6 h. Untreated and H_2_O_2_-treated (4 and 8 mM) parasites were taken as negative and positive controls, respectively and DNA fragmentation of isolated genomic DNA was done as described earlier^24,64^. Briefly, treated cells were harvested by centrifugation and the pellet was suspended in 200 µl of PBS buffer. Thereafter cells are lysed with 200 µl of lysis buffer from the QIAamp DNA Mini kit containing proteinase K (200 μg/ml), and RNase A (40 μg/ml) for 3 h at 37 °C. The degraded DNA was separated by addition of phenol/isopropanol/chloroform and centrifugation at 12,000*g* for 15 min at 4 °C. Aqueous layer containing the genomic DNA was precipitated by sodium acetate (pH 5.0) and ice cold ethanol. Mixture was centrifuged at 15,000*g* for 15 min and then pellet was dissolved in 30 µl of TE (10 mM Tris–HCl pH 7.5, 1 mM EDTA) buffer. Sample were loaded on 1.5% agarose gel and visualized under UV illumination after ethidium bromide staining using 1 kb and 100 bp ladder as size markers.

### FACS analysis for apoptosis and necrosis

Annexin V-FITC (AV)/propidium iodide (PI) binding of parasites was assessed using the AV Apoptosis Detection Kit. Briefly, promastigotes (1 × 10^6^ cells/ml) were treated with CA (173 µM and 346 µM) for 6 h. Untreated cells were taken as the negative control. Cells were harvested, washed, and dissolved in 1 ml PBS followed by mixing with the sequential addition of 5 μl PI, 20 μl of binding buffer, and 5 μl of AV. After 15-min incubation, cells were analyzed by a FACS Calibur flow cytometer (Becton Dickinson, CA, USA)^24^.

### RNA extraction, cDNA synthesis, and semiquantitative-PCR

The freshly grown mid-log phase parasites were treated with CA (173 μM and 346 μM). AmB (0.05 μM) treated cells were taken as the positive control, while untreated cells were taken as the negative control. After 6 h of treatment, cells were processed for RNA isolation using the TRIZOL method, followed by cDNA synthesis, semi-quantitative PCR, and analysis as described^24^. Genes (MevK, HMGCR, FPPS, TryR, APX, and α-tubulin) were amplified by PCR using cDNA as a template. The PCR mixture (25 μl) contains 0.6 μM of forward and reverse primer, 0.5 mM of each dNTP, 2 mM MgCl_2_, 0.5 μg of synthesized cDNA, and 1 μl Taq polymerase. The sequence of PCR primers were shown in Table 1 of Supplementary information. The PCR was done for 28 cycles where each cycle had denaturation at 95 °C for 30 s, annealing (ranging from 55 to 64 °C) for 30 s, and extension at 72 °C for 45 s. The fold change in gene expression was calculated by densitometric scanning using SYNGENE (G box) Genesys software.

### Molecular docking and molecular dynamics analysis

The crystal structure of *L.* *major* MevK with ligand R-mevalonate was downloaded from the PDB database (PDB ID 2HFU). The methodology for preparing enzyme and ligand for docking and molecular dynamics (MD) studies were adopted as described^65^. The enzyme was prepared using the protein preparation wizard of Schrodinger’s software. It was done through the operation of assigning bond orders, adding hydrogens, missing side chains, and loops, and deleting waters beyond 5.0 Å from the substrate R-mevalonate. Chain A with R-mevalonate selected and optimized for H-bond assignment. Energy minimization was done through the OPLS-2005 force field. The binding site encompassed the ligand, which was observed in the crystal structure of MevK. The ligand, CA, was obtained from a 2D sketcher using the LigPrep module. The 20 Å grid was generated around the R-mevalonate using a receptor grid generator. The CA and R-mevalonate were docked using Glide. The conformations of SP docking were subsequently submitted to XP docking. The resulting conformations were analyzed based on the interaction between energy and pose.

The docking pose of CA is selected for MD simulation based on the best fit of the pose in the active site pocket. R-mevalonate co-crystal pose was used for MD simulation. The active site-ligand complex was first solvated with the TIP3P water molecules around 10 Å of the active site edge. The charge on the system was neutralized by adding counter ions. The system was first equilibrated and then submitted for a 100 ns MD production run. The MD has been performed on HP system configuration of Intel Xenon CPU speed of 2.3 GHz, 12 cores with GPU Quadro K4200 by using Desmond (version 4.2, D.E. Shaw Research, New York, NY, 2015). Further, the Prime MM-GBSA has been performed to calculate the ligand-binding energies of ligands with the active site. MMGBSA calculation was done using the Prime MM-GBSA tool of Schrodinger with VSGB solvation parameter and OPLS2005 force field^27^.

### Cloning, expression, and purification of recombinant LD-MevK

MevK gene was amplified by PCR using forward (5′ GTGTGGATCCATGCCAAAGCCCGTCAAGAG 3′) and reverse primer (5′ GTGTAAGCTTTTACAGGTTTGACGCGGTGG 3′) with 100–150 ng of LD genomic DNA as a template in a 100 µl of PCR reaction. The restriction enzyme sites in primer are underlined. The PCR was done for 35 cycles where each cycle had denaturation at 95 °C for 30 s, annealing at 54–60 °C for 45 s, and extension at 72 °C for 1 min. PCR product was gel extracted and digested along with pET28a vector with restriction enzymes BamHI and HindIII. The ligation, transformation, and selection of clones were done using standard procedures using *E. coli* DH5α as competent cells. After successful isolation of clones from DH5α cells by restriction digestion of plasmids, the same plasmids were transformed in BL-21 (DE3) cells for protein expression. The BL-21 (DE3) cells containing the MevK clone in pET28a were induced with 0.1 mM IPTG for 4 h at 37 °C. The pellet containing 100 ml induced culture was sonicated and the supernatant containing soluble MevK was used for purification using Ni-NTA agarose columns. The 5 ml of supernatant containing overexpressed MevK was purified with 0.5 ml of Ni-NTA columns which was equilibrated with 50 mM Tris–HCl buffer (pH 7.5), 300 mM NaCl and 5 mM imidazole. The bound MevK proteins are eluted in presence of 400 mM imidazole in different fractions in presence of same buffer. The fractions of purified MevK were pooled and dialysed in 10 mM of Tris–HCl buffer (pH 7.5) containing 50 mM NaCl and 10 mM MgCl_2_ for overnight at 4 °C. The protein content in the dialysed proteins were measured by Bradford assay and, further, used for enzymatic studies and western blot. Western blot was performed using Anti-6xHis as primary and anti-mouse IgG-HRP as the secondary antibody to check the over-expression of LD-MevK. The mutation Y167F was created using the following primer sequences: forward primer-5′GACTCATTTCGTTCCGTCGC3′, reverse primer 5′GCGACGGAACGAAATGAGTC3′. The single base change which converts Y167 of MevK to F167 is underlined. For site-directed mutagenesis, the WT plasmid in pET28a was used along with these primers, and the PCR was done using high fidelity Taq DNA Polymerase. The initial denaturation was at 98 °C for 30 s followed by 30 cycles consisting of 98 °C denaturation for 10 s, 60–66 °C annealing for 30 s, and extension at 72 °C for 3.5 min. The PCR was gel extracted, digested with Dpn1 restriction enzyme for 1 h, and then transformed in DH5α cells as described above. The mutation was confirmed by sequencing. Mutant Y167F protein was purified as described above.

### Homologous expression of MevK in *L. donovani*

The Ld-MevK ORF was also cloned into pLpNeo2 using restriction enzymes BamHI and XhoI and then transfected into promastigotes by electroporation at 25 μF, 1500 V (3.75 kV/cm) with a gap of 10 s between pulses using Gene Pulser X-Cell (Bio-Rad)^31^. Parasites were transfected with pLpneo2 empty vector alone as a control. Transfectants were grown and selected with increasing concentrations of neomycin (10–100 µg/ml) and finally maintained in 100 μg/ml of neomycin. Over-expression of the MevK gene was measured by semi-quantitative PCR. Further, both transfected and non-transfected LD cells were treated with CA (70–700 μM) to measure the change in IC_50_ values, if any.

### MevK activity assay

The MevK assay was carried out with slight modification as described earlier^30^. The activity of the recombinant LD-MevK assay was assessed in a buffer containing 100 mM glycine, 25 mM KCl, 4 U LDH, 4 U pyruvate dehydrogenase, 1 mM phosphoenol pyruvate, 5 mM ATP, 5 mM MgCl_2_, R-mevalonate (0.5–8 mM), 160 µM β-NADH and 30–50 μM Ld-MevK at 37 °C for 10 min. The enzyme and β-NADH were added last in the reaction. The oxidation of NADH at 340 nm was measured using a microplate reader. The activity assay at different pH (3–11 using glycine–NaOH buffer), different temperatures (25–65 °C), and with different salts (MgCl_2_, CaCl_2_, ZnCl_2_, MnCl_2_, CuSO_4_, and NaCl) were done using 4 mM R-mevalonate as substrate. Further, enzyme inhibition studies were carried out using CA (2.5–100 μM), and 100 µM of 4m-CA, 1m-CAE, and monocaprylin. The enzyme and inhibitors were incubated together in the reaction mixture for 10 min followed by the addition of β-NADH. The Michaelis–Menten equation with a non-linear least square algorithm in Graph-Prism 5.0 was used to calculate kinetic parameters, K_m_ and V_max_.

### Circular dichroism spectrometry

Circular dichroism measurements were carried out on a JASCO 1500 circular dichroism spectrophotometer. 150 µl 40 µM of LD-MevK in buffer A (0.1 M Tris–Cl, 5 mM MgCl_2,_ and 5% glycerol, pH 7.4) was taken in a micro cuvette of pathlength of 1 mm and scanned over the wavelengths of 200–300 nm at a scan rate of 100 nm/min with three accumulations. The same was repeated with the addition of 1 µl of 20 mM of R-mevalonate and CA separately.

### Isothermal calorimetry

Isothermal calorimetry (ITC) was performed on a Micro Cal iTC_200_ (GE Healthcare). For the titration, buffer A was used. 200 µl of 40 µM LdMevK was loaded into the sample cell and 40 µl 1 mM substrate (R-mevalonate) or inhibitor (CA) solution was taken in the injection syringe separately. Then, the titration was performed according to the following setup: rotation speed 600 rpm, temperature 37 °C, initial delay 60 s, injection volume 2 µl except the first one (0.4 µl), interval time between two injections 120 s, total 20 injection. Then the data were fitted with instrument integrated software based on Origin (Origin Lab Corporation, USA) to obtain the binding constant (K), no of binding sites (n), change in enthalpy (ΔH), and change in entropy (ΔS).

### LC-HRMS based proteomics analysis for CA treated parasites

Promastigotes were grown at a density of 2 × 10^6^ cells/ml for 48 h then and treated with CA (173 and 346 µM) for 6 h. Treated and untreated parasites (1 × 10^8^) were harvested, washed thrice with PBS finally, and then finally lysed in 300 μl of lysis buffer (50 mM Tris–HCl, 100 mM NaCl, 0.1% Nonidet P40, and 0.15% SDS, pH 7.2) in the presence protease inhibitor cocktail of 1 mg/ml. The mixture was incubated for 30 min at 37 °C with shaking. Protein lysates were centrifuged at 12,000*g* for 15 min. The supernatant was collected and precipitated with 4-fold ice-chilled acetone overnight. The pellet was air-dried in 100 µl of PBS. Approximately 100 µg protein (~ 25–30 µl) from untreated, 173 and 346 µM CA-treated samples were reduced with 10 mM DTT for 1 h at 37 °C followed by alkylation with 25 mM of iodoacetamide (IAA) for 1 h at room temperature in the dark. Excess IAA was quenched by adding 10 mM-DTT and incubated at room temperature for 20 min. Sample digestion was performed overnight at 37 °C by addition of 50 mM of 120 µl ammonium bicarbonate with 2 µg trypsin. After digestion, 5 μl of 0.25% formic acid was added to the mixture to hydrolyze the trypsin and the entire digestion mixture is dried in a vacuum concentrator and reconstituted in 0.1% formic acid before mass spectrometry analysis.

Reconstituted samples were subjected to mass spectrometric analysis using Orbitrap Exploris 240 mass spectrometer (LTQ-XL, ThermoFisher Scientific) after chromatographic separation and peptide fractionation through a C18 easy spray nano column (3 µm, 100 Å) by nano-LC (Easy-nLC1000). The injection volume was 2 µl for each sample with a spray rate of 300 nl/min. Peptides were eluted using a 150-min dual gradient of solvent A and B (solvent A: 0.1% formic acid in water; solvent B: 0.1% formic acid and 80% acetonitrile in water). The gradient profile was set with solvent B as follows: 5–20% for 60 min, 20–60% for 40 min, 60–95% for 20 min, 95% for 10 min, and 5% for 20 min. The scan range for Orbitrap was from 350 to 2000 (m/z) with a minimum of three peaks and a resolution of 60,000. Ionic fragmentation was done by the collision-induced dissociation (CID) method. Protein identification was performed by Thermo Proteome Discoverer version 1.4.0. MS/MS spectra were matched against MASCOT with the assistance of a Percolator. Static modification was set for N-terminal acetylation and carbamidomethylation of cysteine. The dynamic modification was set for methionine (oxidation) and the minimum missed cleavage number was set at two. Swissprot was used as a reference software for protein identification.

### Statistical analysis

The statistical analysis was done by one-way and two-way ANOVA using GraphPad Prism software (version 5.00; GraphPad Software Inc., La Jolla, CA, USA). The results were measured as mean ± SD of at least three independent experiments. The results were shown as approximate mean values. Differences between group data (especially between CA and control) were considered statistically significant and highly significant when P < 0.05 and P < 0.001, respectively.

## Supplementary Information

Supplementary Information.

## Data Availability

All data and material, included in this manuscript, is not published or submitted anywhere else. Proteomics data are available via ProteomeXchange repository with Accession no PXD034560 and 10.6019/PXD034560. The Reviewer account details: Username: reviewer_pxd034560@ebi.ac.uk. Password: Grwkc5UB. This details can be used to review the submitted data with Accession no PXD034560.
