# Supplementary material for: Mechanistic insight into the role of mevalonate kinase by a natural fatty acid-mediated killing of Leishmania donovani
Source: Sci Rep. 2022 Sep 30;12:16453. doi: 10.1038/s41598-022-20509-9 (PMC9525708; doi:10.1038/s41598-022-20509-9)
Supplement: Supplementary file 1 — Supplementary Information. [file 41598_2022_20509_MOESM1_ESM.docx]

**Supplementary Information**

A


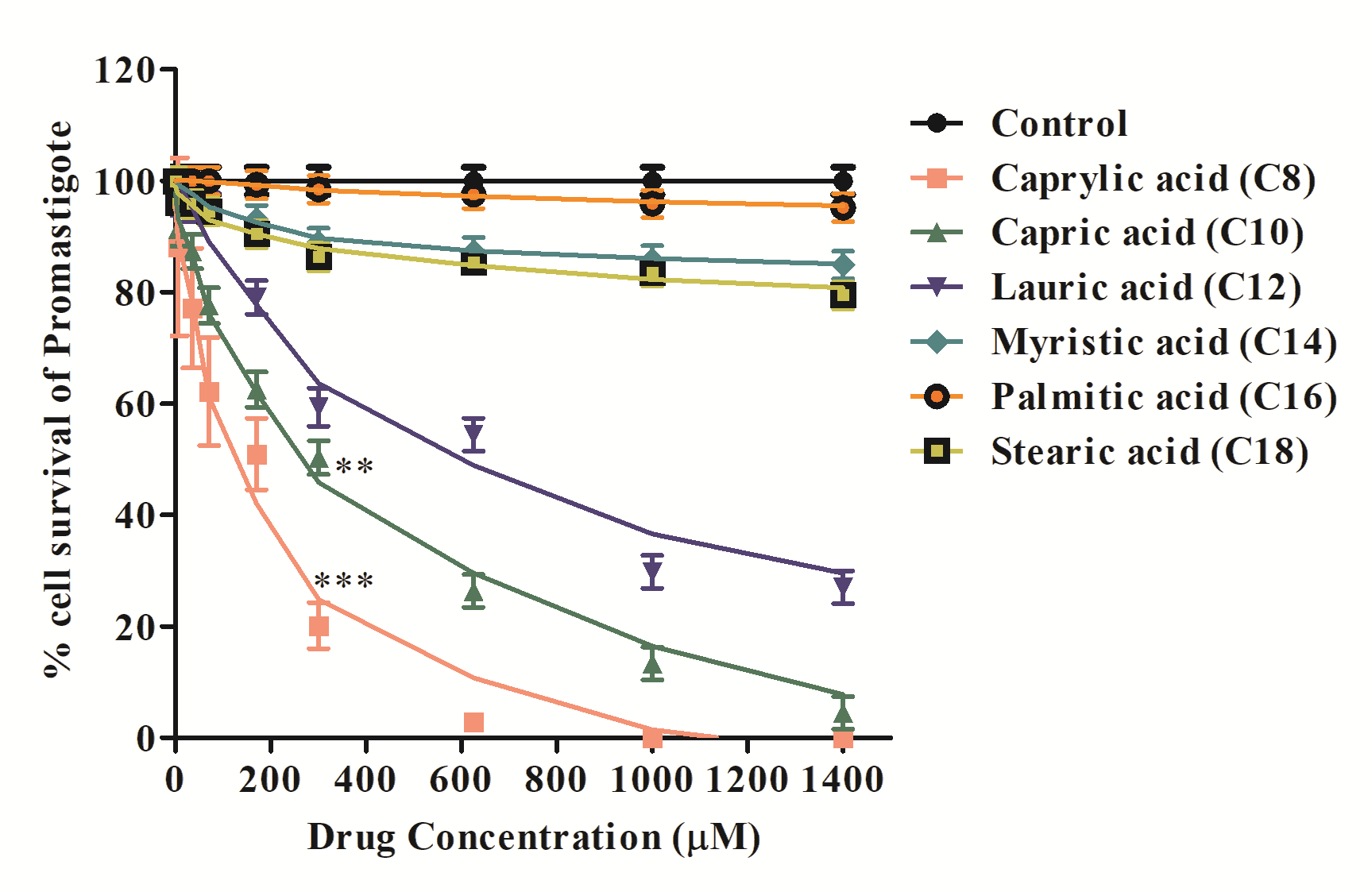


**SI-1.**Saturated medium chain fatty acids with chain length (C8-C18) were tested in vitro for antileishmanial efficacy (A).


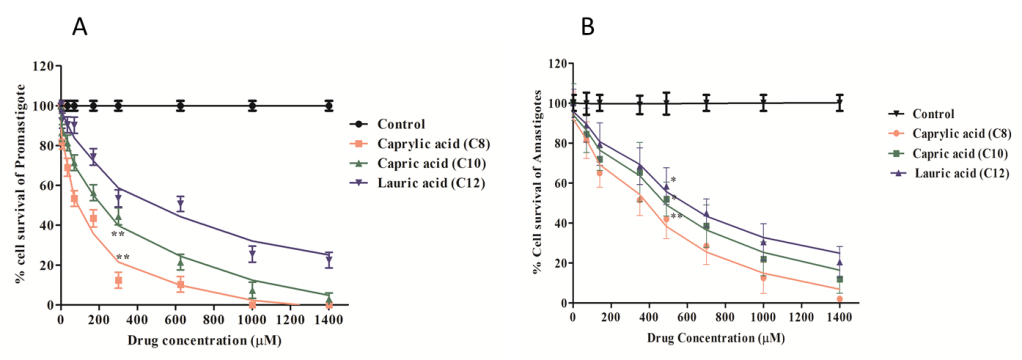


**SI-2.**The antileishmanial effect of CA was evaluated on both form promastigotes (A) and amastigotes (B) of leishmania after 72 hrs of treatment.


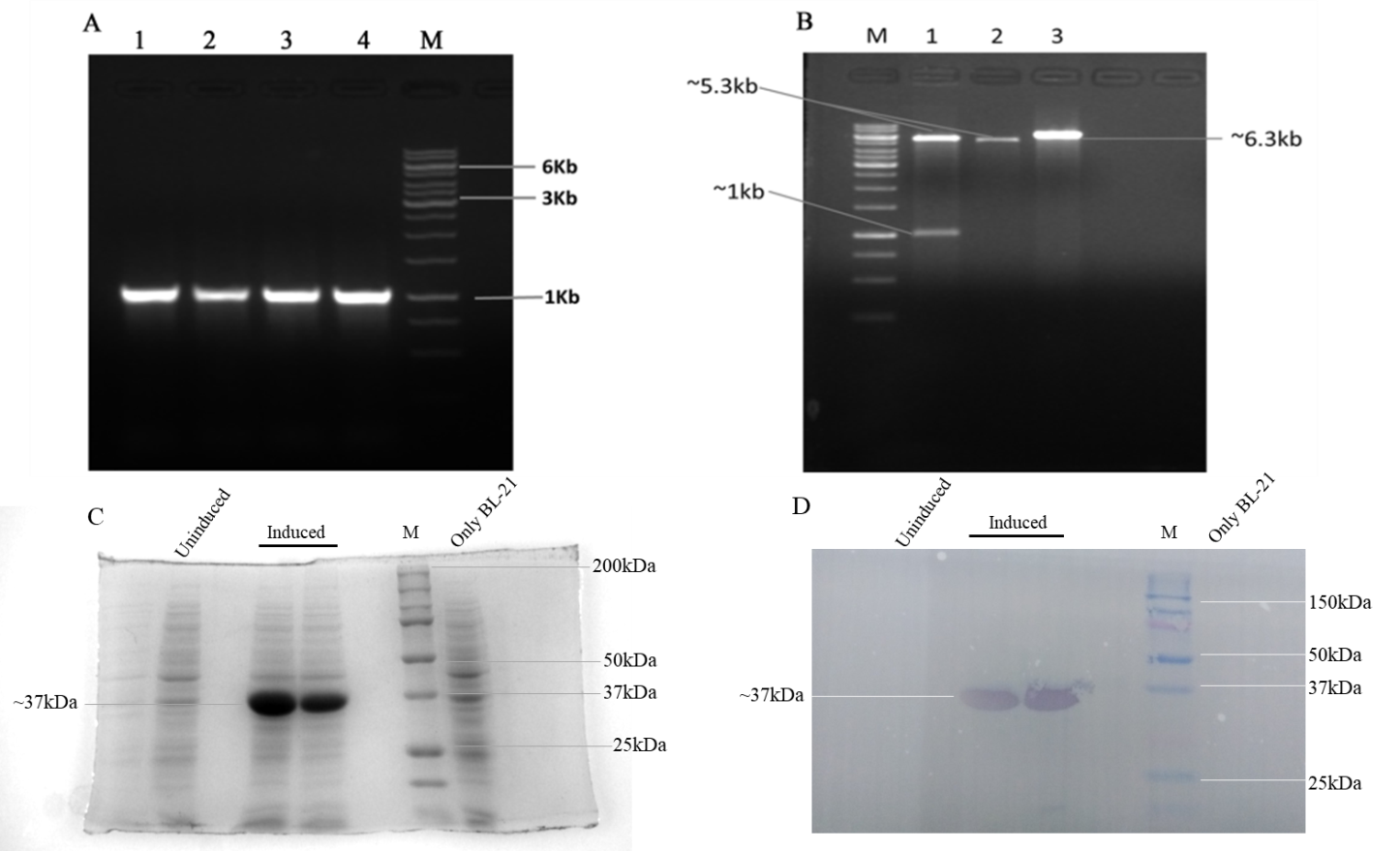


**SI-3.**LDMevK cloning and protein expression was performed using pET28a plasmid in the *E. coli*DH5α strain.Analysis of PCR product of ~1Kb at different annealing temperaturesby 1% agarose gel electrophoresis (A). 1% Agarose gel of restriction enzyme digested cloned plasmid (B). 10% SDS-PAGE run to check protein expression which was carried out by induction of IPTG with desired band at ~37kDa (C) Confirmation of protein expression bywestern blot using anti-His antibody (D).


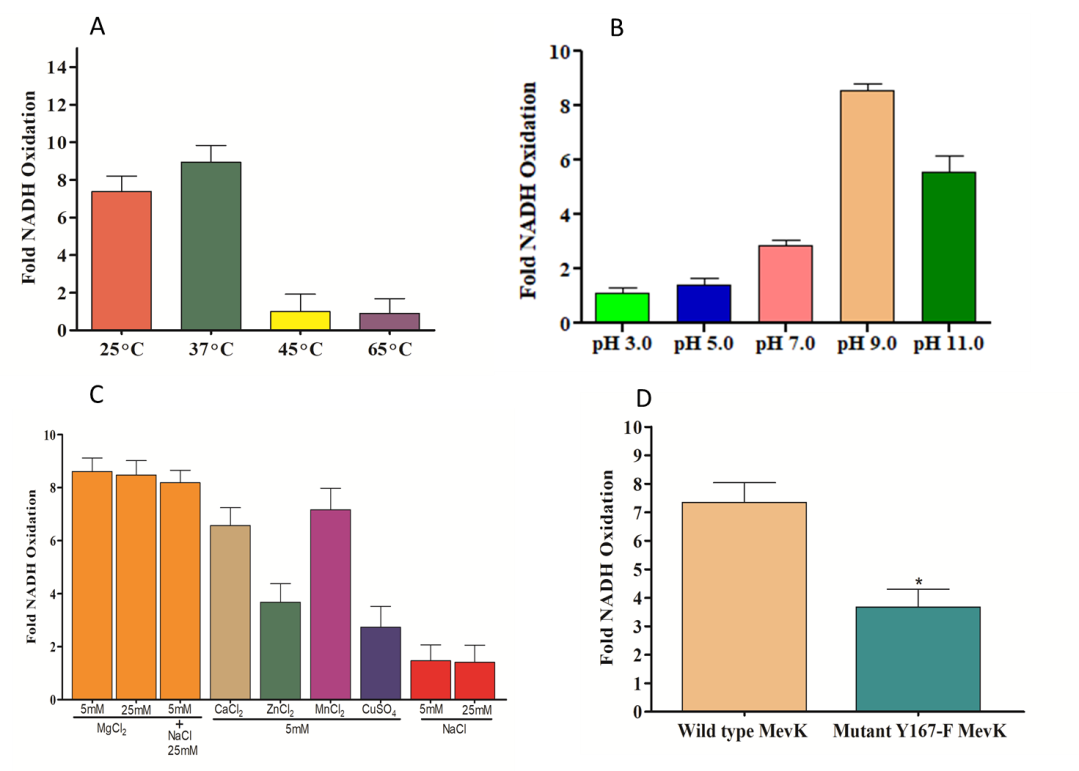


**SI-4.**LD-MevK enzymeactivity at different temperatures (A), pH (B), and in presence of different divalent/monovalent cations (C).Enzyme activity of wild-type and mutant (Y167F) MevK (D).


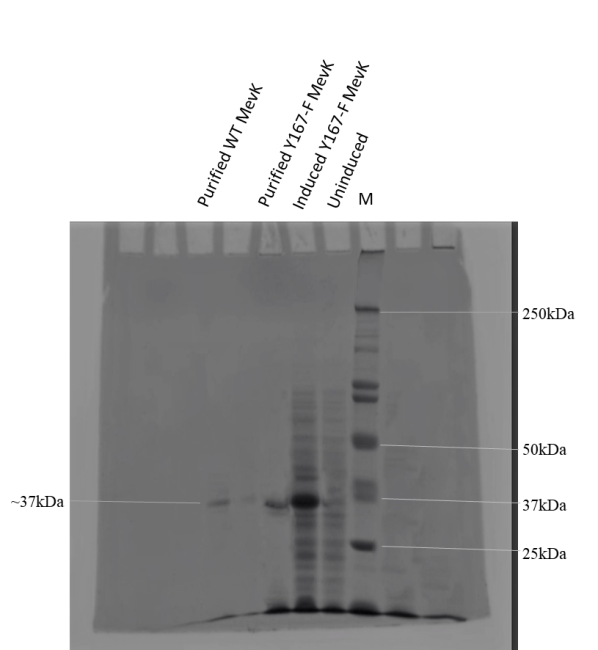


**SI-5.**10%SDS-PAGE analysis of purified WT and Y167F mutant MevK.

**
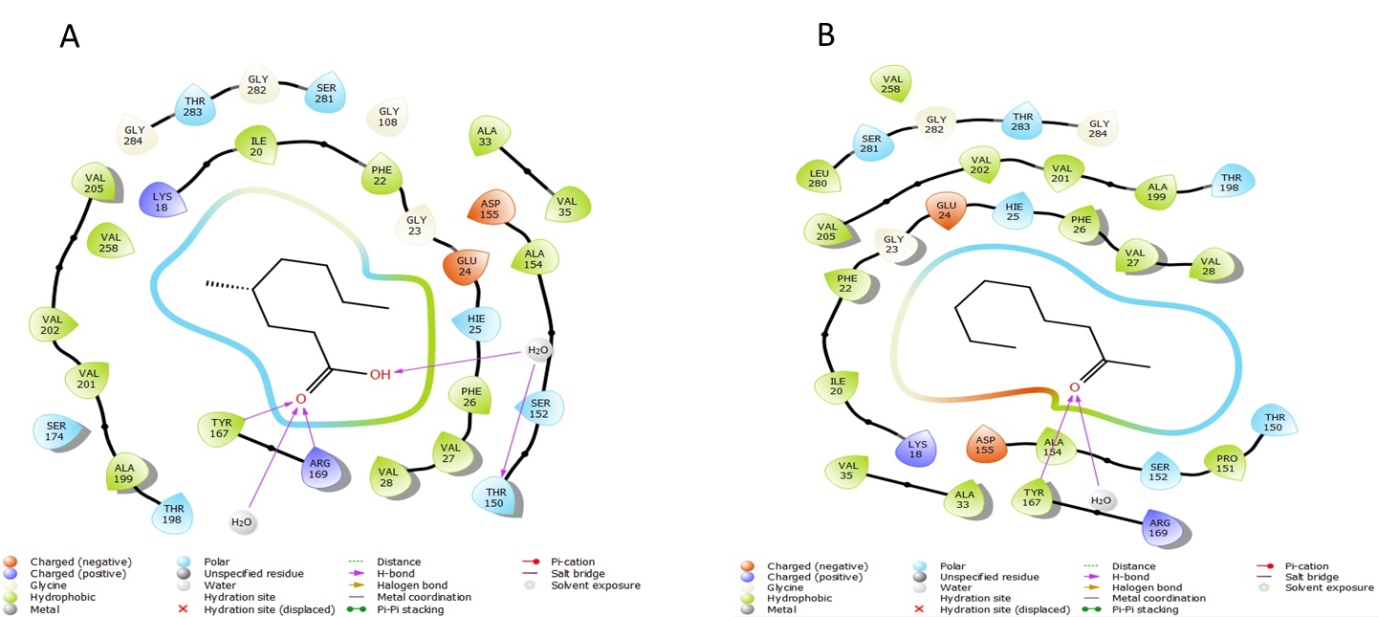
**

**SI-6.**Docking study with CA analogues 4M-CA and 1m-CAE**. It** shows a 2D interaction diagram of MevK+4m-CA (A) and MevK+ 1m-CAE (B)

**Table 1: List of Primer for Semiquantitative PCR**

| Sr.No. | Name | Oligonucleotide sequence |
| --- | --- | --- |
| 1 | MevK_F | TGGCGGACTCATTTCGTACC |
| 2 | MevK_R | TTCATCAGCTGCCCTAACCG |
| 3 | HMGCR_F | ATCGCCATTCGTCGTGAGAT |
| 4 | HMGCR_R | GAGCACCGCGGTGCGCACTC |
| 5 | FPPS_F | ACTACCACGATTGGTCAGCT |
| 6 | FPPS_R | CCAGTAGGTGTAGTAGGCGG |
| 7 | APX_F | GGGTGGATGCCAAGGATGGC |
| 8 | APX_R | ACCCAATCCTCATCGAGCAA |
| 9 | TryR_F | GGCGAAGAACTACGAAACCG |
| 10 | TryR_R | ACGCTCTGGATGATCTCAGG |
| 11 | α-tubulin_F | GCGATTGCGACGATCAAGAC |
| 12 | α-tubulin_R | GGTCGAGTTGGCAATCATGC |
| 13 | MutMevK[Y167- F]- F | 5’GACTCATTTCGTTCCGTCGC3’ |
| 14 | MutMevk [Y167-F]- R | 5’GCGACGGAACGAAATGAGTC3’ |

**Table 2: List of proteins/peptide with fold changes in expression after Caprylic acid treatment**

| **Gene ID or accession ID** | **Description** | **Unique peptide** | | **Down-regulation** | | **Up-regulation** | |
| --- | --- | --- | --- | --- | --- | --- | --- |
|  |  | **1x** | **2x** | **1x** | **2x** | **1x** | **2x** |
| A0A504XW29 | Acetyl-coenzyme A synthetase | 16 | 19 | 2.7 | 2.2 | - | - |
| A0A3S5H5H0 | Very-long-chain (3R)-3-hydroxyacyl-CoA dehydratase | 7 | 2 | 1.4 | 6.64 | - | - |
| I7CKJ5 | 3-hydroxy-3-methylglutaryl coenzyme A reductase | 9 | 2 | - | - | 1.25 | 1.82 |
| **A0A504WXX7** | **Mevalonate Kinase** | 4 | 6 | 2.38 | 6.62 | - | - |
| E9BS25 | Arginase | 5 | 5 | No change | 1.4 | - | - |
| Q95PT6 | Trypanothionereductase | 1 | 1 | 2.4 | No change | - | - |
| A0A3S5H833 | Cysteine synthase | 11 | 7 | No change | 1.85 | - | - |
| E9AGB5 | Ornithine_decarboxylase | 2 | 1 | - | - | 1.3 | 1.4 |
| A0A504XJJ4 | Glutathione synthase | 7 | 5 | 1.6 | 1.26 | - | - |
| A4HWN3 | Phosphomevalonate kinase | 2 | 3 | - | - | 1.02 | 1.1 |
| A0A3S7WRP1 | LeucylAminopeptidase | 10 | 13 | 2.9 | 1.8 | - | - |
| A0A075FJL9 | Heat shock protein 70 | 1 | 1 | 5.9 | 1.5 | - | - |
| A0A504XG13 | Leishmanolysin | 2 | - | 3.8 | - | - | - |
| A0A3Q8IGF4 | Trypanothionesynthetase | 2 | 1 | No change | 1.57 | - | - |
| E9BJR1 | DNA topoisomerase | 1 | 1 | - | - | 2.56 | 3.28 |
| A4HVU0 | Squaleneepoxidase | 1 | 3 | No change | No change | 1.05 | 1.09 |
| C5MLW7 | Lanosterol 14-alpha demethylase | 3 | 5 | - | - | 2.4 | 3.85 |


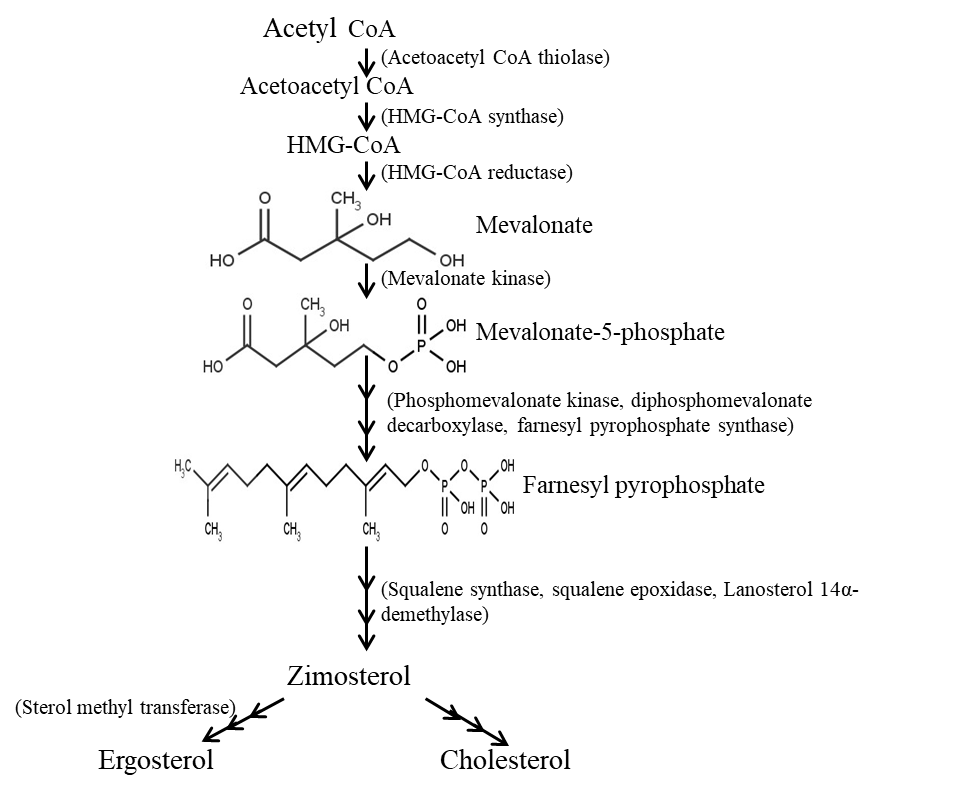


**SI-7:** Ergosterol/Cholesterol biosynthesis pathway of *L.donovani* with role of mevalonate kinase indicated.

**Original unprocessed images**

**
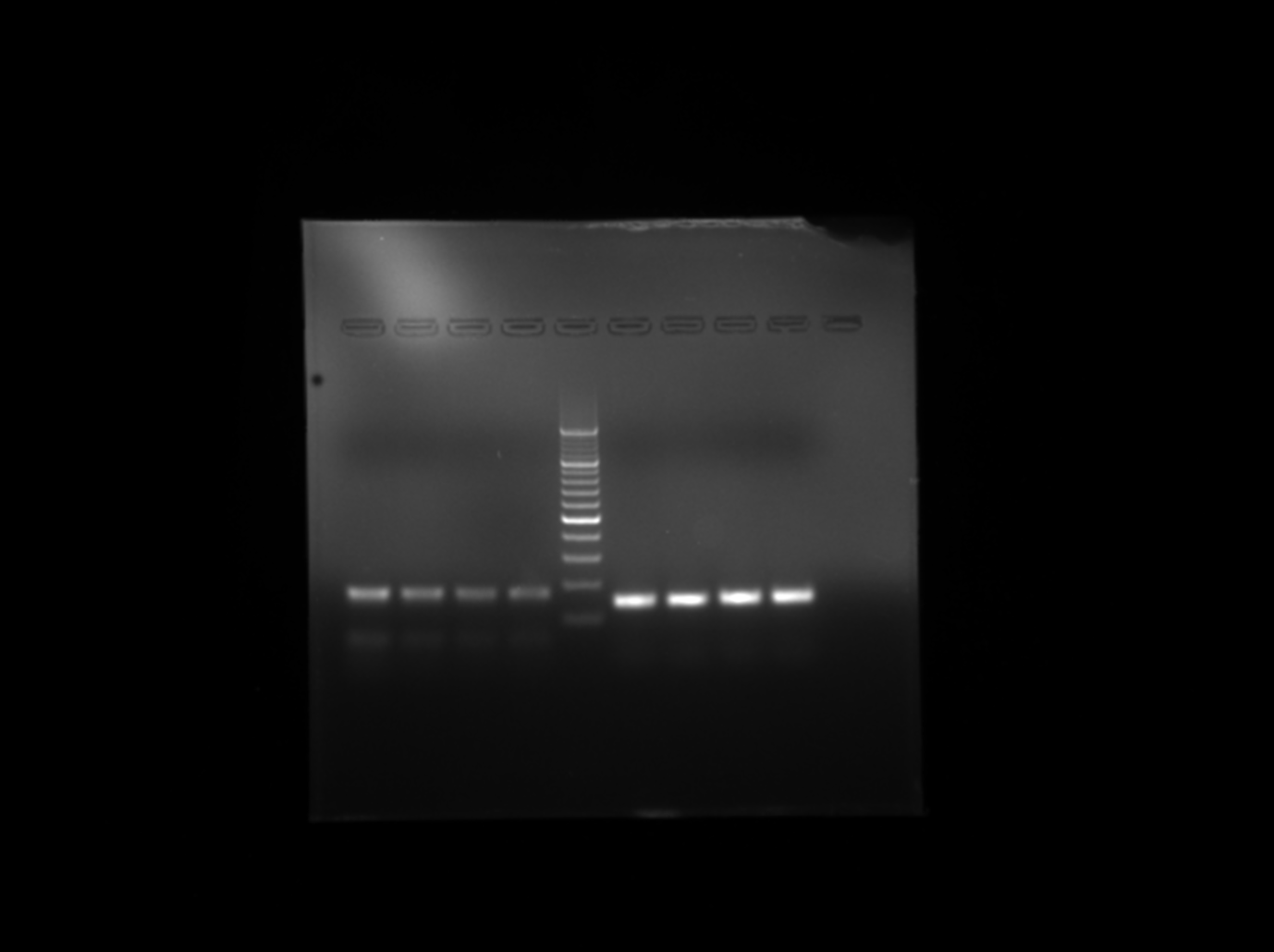
**

TryR

α-tubulin

Fig 4C gene expression study by semiquantitative PCR for gene TryR and α-tubulin


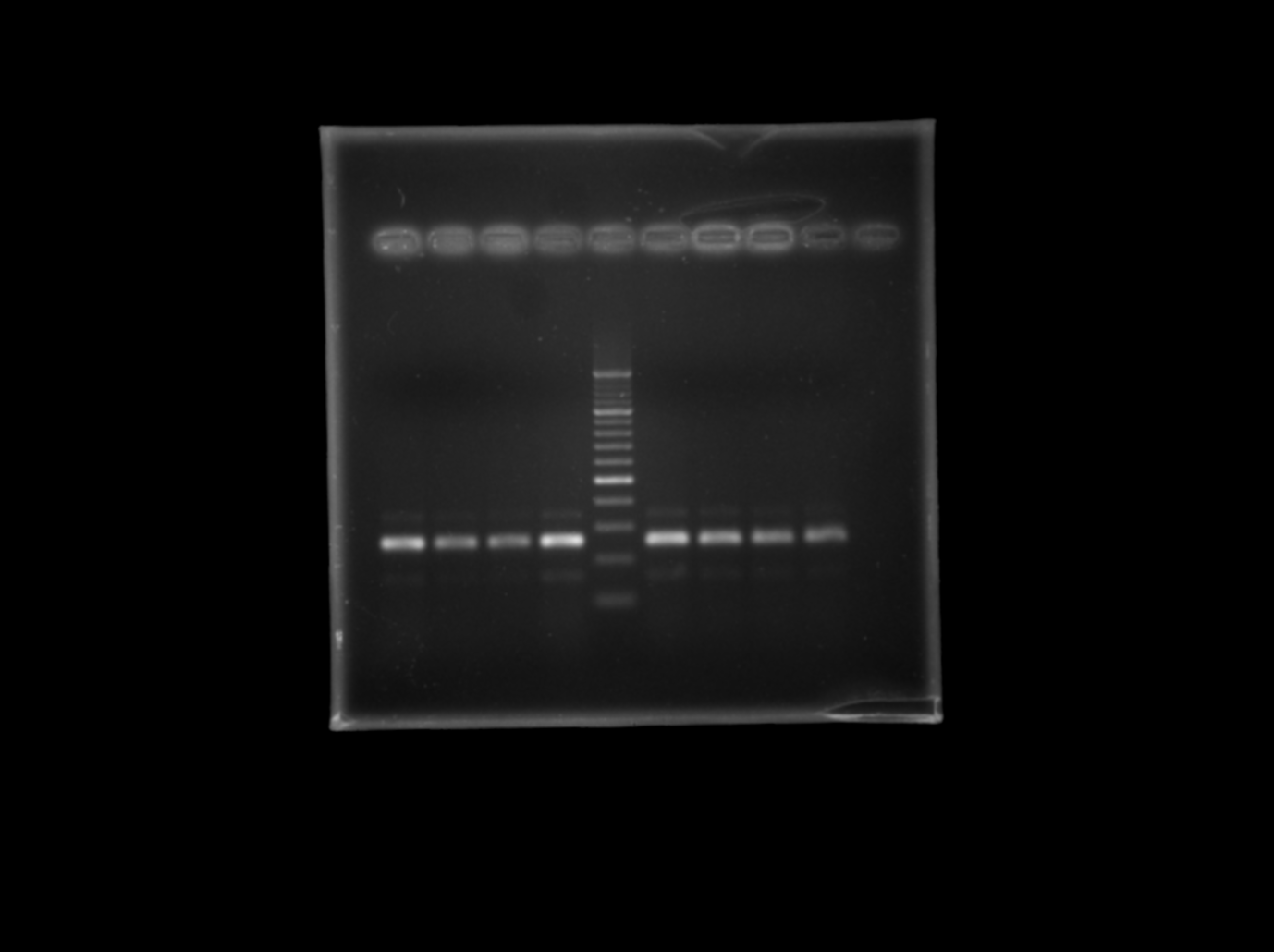


APX

MevK

Fig 4C gene expression study by semiquantitative PCR for gene MevK and APx


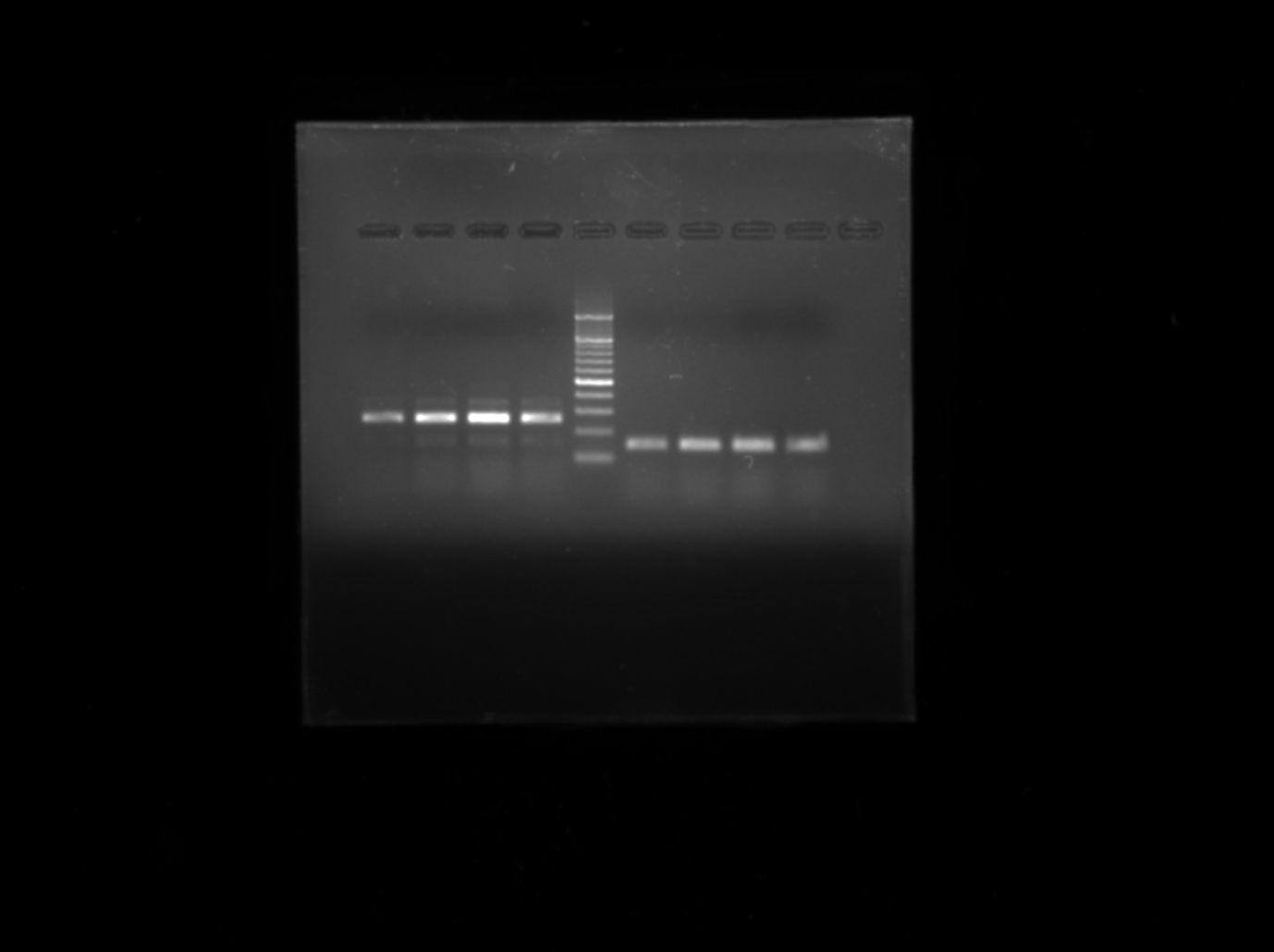


HMGCR

FPPS

Fig 4C gene expression study by semiquantitative PCR for gene HMGCR and FPPS.


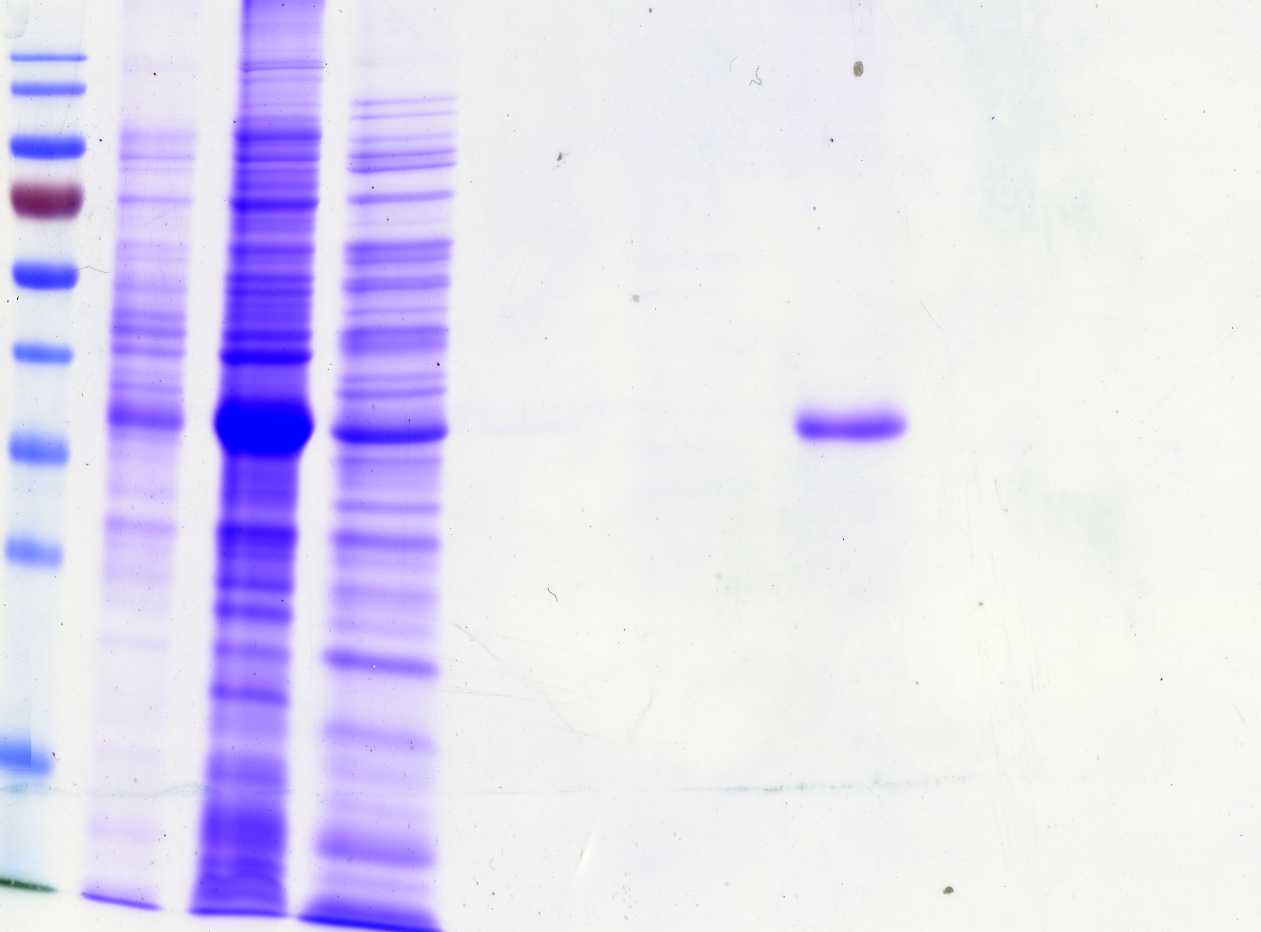


Fig 7A 10% SDS-PAGEanalysis of purified LD-MevK


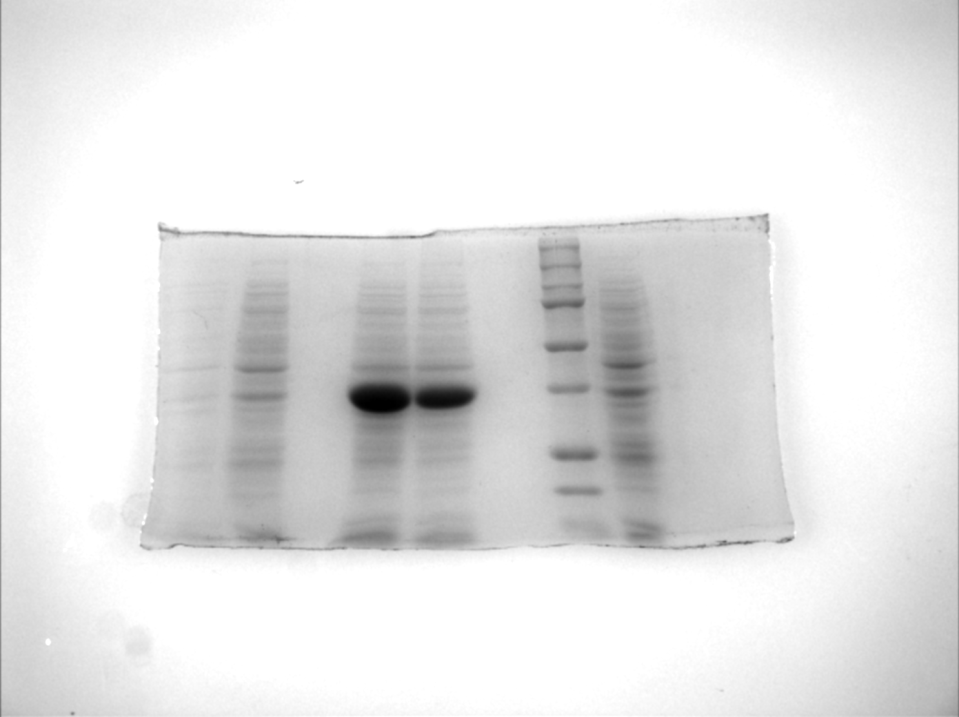


**Fig SI-3C** 10% SDS-PAGE run to check protein expression which was carried out by induction of IPTG with desired band at ~37kDa.


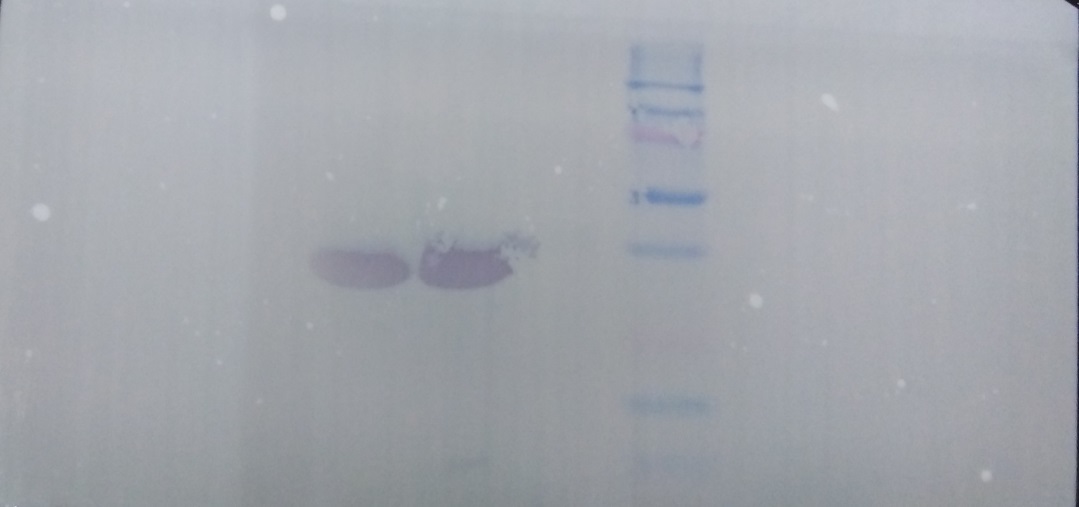


Fig SI-3D Confirmation of protein expression bywestern blot using anti-His antibody


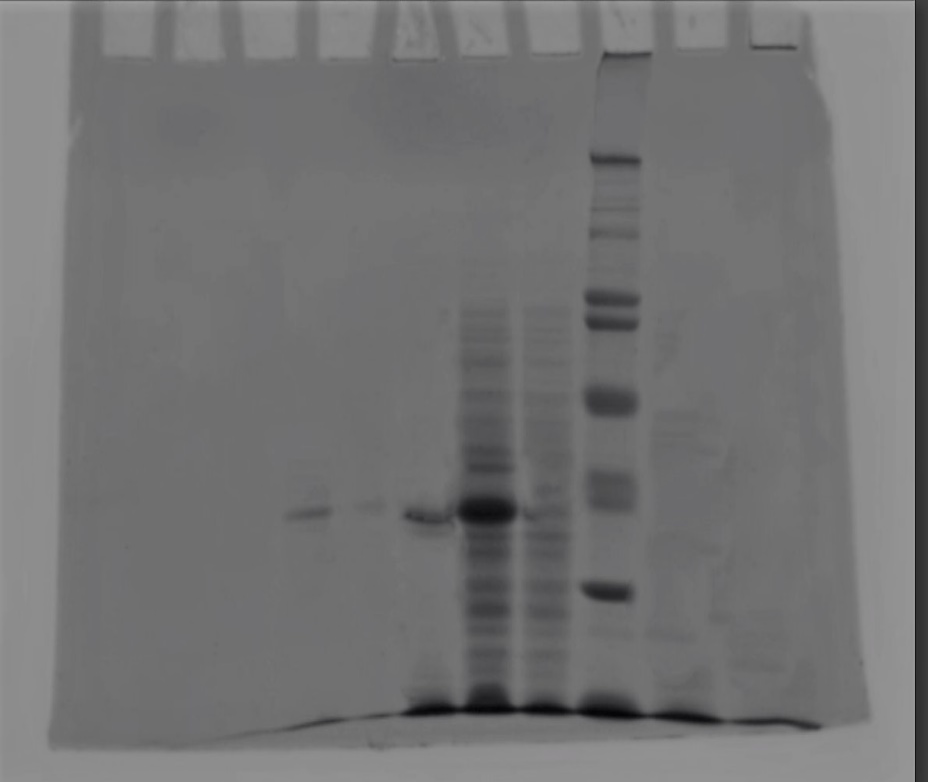


SI-5 10%SDS-PAGE analysis of purified WT and Y167F mutant MevK
